# Acetonitrile based single step slot-die compatible perovskite ink for flexible photovoltaics†

**DOI:** 10.1039/c9ra06631d

**Published:** 2019-11-15

**Authors:** Daniel Burkitt, Richard Swartwout, James McGettrick, Peter Greenwood, David Beynon, Roberto Brenes, Vladimir Bulović, Trystan Watson

**Affiliations:** SPECIFIC, College of Engineering, Swansea University Bay Campus SA1 8EN Swansea UK daniel.burkitt@swansea.ac.uk t.m.watson@swansea.ac.uk; Research Laboratory of Electronics, Massachusetts Institute of Technology 77 Massachusetts Avenue Cambridge MA 02139 USA

## Abstract

The demonstration of photovoltaic devices with high power conversion efficiencies using low cost perovskite materials hints at the possibility of dramatically lowering the cost of solar energy. Key to further exploiting the potential of these materials is developing rapid processing techniques that can be used to deliver lower cost high throughput manufacture. This work details the development of low viscosity rapid drying perovskite formulations designed to give high quality solar films when slot-die coated on flexible roll-to-roll compatible substrates. A single step slot-die compatible perovskite ink based on an acetonitrile/methylamine solvent system utilizing a chloride additive is developed, resulting in large area perovskite films from slot-die coating under ambient conditions. The drying conditions for the perovskite film are optimized and fast (<10 min), low temperature (<120 °C) drying of slot-die coated films on flexible substrates are demonstrated and result in high performance devices.

## Abstract 2

Low viscosity rapid drying perovskite formulations designed to give high quality solar films when slot-die coated on flexible roll-to-roll compatible substrates are developed .

## Graphical abstract

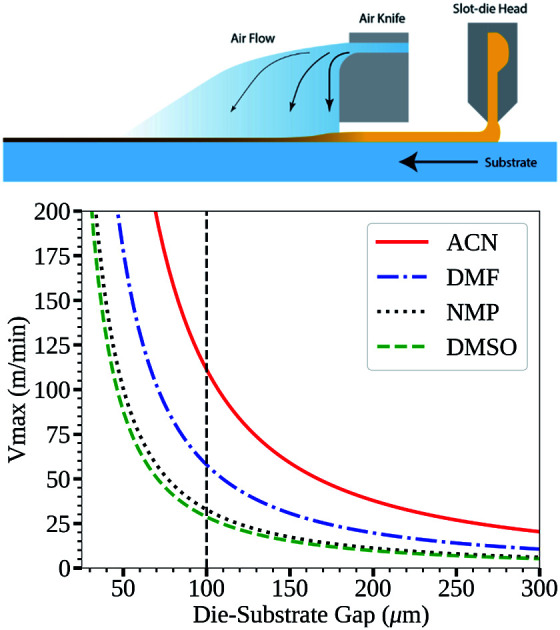

## 1 Introduction

A large portion of the cost of conventional photovoltaic solar energy comes from the bill of materials for the module, the high cost of the absorber material, and the speed of manufacturing relative to required capital expenditure (CapEx).^1,2^ To further lower the cost of solar energy, which is required for high solar penetration in modern grids, research has been directed at finding lower cost absorber materials that give high power outputs and are processed easily. This work explores high throughput processing of perovskite materials.

Perovskite based photovoltaic materials have gained a great deal of research interest due to the high certified efficiencies, low cost of precursor materials, and low cost and low temperature manufacturing routes.^3–5^ The ability to solution process these materials at low temperature and incorporate into flexible device stacks gives the possibility of using high throughput manufacturing processes, such as roll-to-roll printing and coating, that could be exploited to further reduce the cost of manufacturing.^3,4^ Flexible roll-to-roll compatible manufacture allows for unique rapid deployment strategies, as demonstrated for organic photovoltaics,^6–8^ that have the potential to reduce installation costs and increase the rate of installed capacity.

Roll-to-roll processing can be used for high throughput manufacture, with line speeds of hundreds of meters per minute used for printing and coating of common products, *e.g.* print media. The solution processability of perovskite materials makes them ideal candidates for deposition using roll-to-roll compatible coating methods such as blade,^9,10^ spray^11^ and slot-die,^12–33^ as well as printing techniques such as screen,^34,35^ inkjet^36,37^ and gravure.^38^

Slot-die coating stands out due to high reported power conversion efficiencies (PCE), high line (substrate/web) speeds, high material utilization through pre-metering, closed atmospheric system and industrial prevalence.^25–27^ In slot die coating, wet film thickness is set by the flow rate of ink delivered to the coating head for a given line speed and head width. Fine control of ink flow gives fine control of the wet film thickness and thus the resulting dry film, with wet films of less than 5 μm and dry films of less than 20 nm possible. For the generally low inertia/capillary fluids used for the fabrication of perovskite solar cells one of the most important limits to slot-die coating speed is the so-called “low-flow limit” and the break up of the downstream meniscus of the coating bead. The slot-die coating operational conditions are driven by the capillary number, as seen in eqn (1). Here, *μ* is the ink viscosity, *V* is the speed of the line (web) and *σ* is the surface tension. There is an upper limit, the low-flow capillary number where flow becomes unstable. This relation is usually determined empirically, as shown in eqn (2). Here, *H* is the die-substrate gap height and *t* is the wet film thickness.1
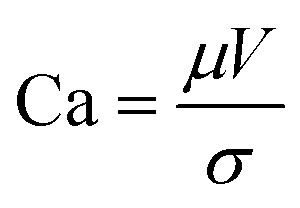
2
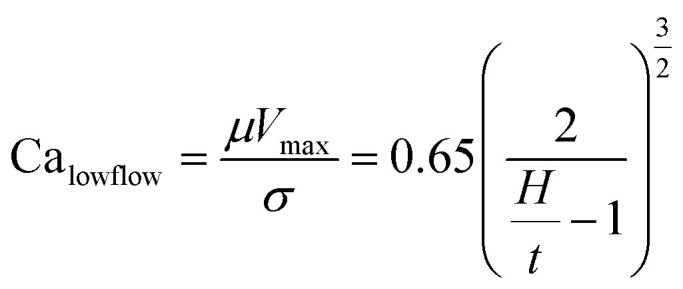


Relating eqn (1) and (2) it becomes clear that the upper limit to manufacturing speed is governed by the rheology of the ink and thickness of the wet film. Crossing this low-flow boundary limit creates non-uniformities such as ribbing, rivulets or discontinuous film formation.^39,40^ In practical applications, gap height is limited to a few tens of micrometers and manufacturing speed is limited by the oven length, in order to accommodate various thermal annealing or curing times. Surface tension is often controlled using surfactants and surface modifiers in order to promote spontaneous or low wetting (<15°) angle conditions between the ink and the substrate. This in turn increases resistance to de-wetting, particle defects and other thin film non-uniformity pathways. Thus, it is ideal to use low viscosity, moderate surface tension and low boiling point solvent systems as it allows for higher coating speeds, substrate wetting, smaller wet films, thinner dry films, faster drying times and faster manufacturing speeds.

Recently, perovskite thin films have been reported using traditional methylammonium lead iodide,^41,42^ as well as higher band gap methylammonium lead bromide^43^ and lower bandgap methylammonium tin lead iodide^44^ using a mixed solvent system of acetonitrile (ACN) and methylamine. This system takes advantage of the complexation ability, and the acid–base nature of amines with transition metals that allows for higher solubility of the lead halide precursor in aprotic solvents.^45,46^ This chemical reaction pathway has also been used to passivate surfaces of perovskite crystals, improve grain growth and redissolve films to heal poor morphologies, but may also be a source of impurities and the reduction of lead(ii) to lead(0) metal.^46^

**Fig. 1:**
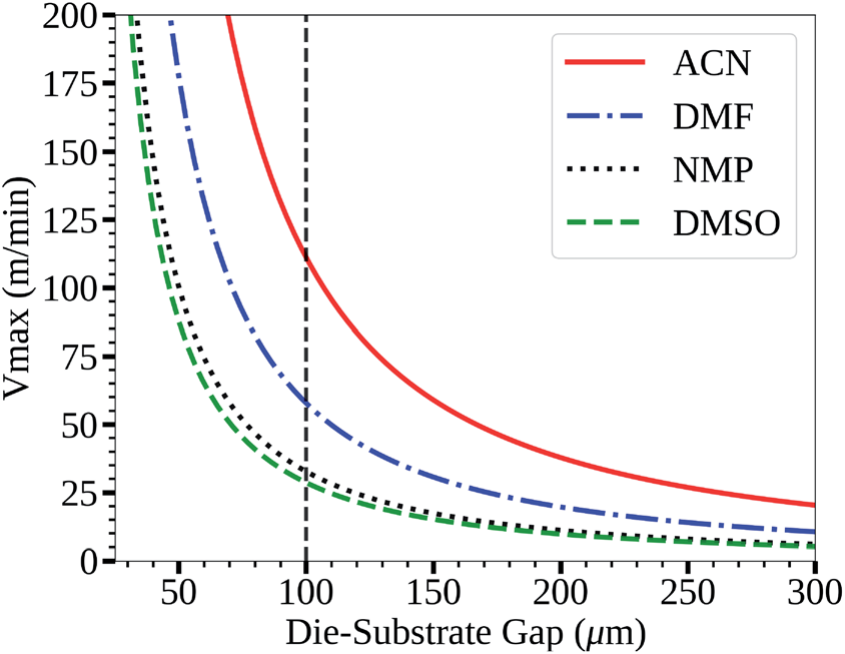
Low flow boundary limit for a 5 μm wet film for common perovskite solvents.

The acetonitrile/methylamine system is interesting for a few reasons: firstly, it utilizes methylamine as the Pb–alkylamide former,^43,45^ thereby reducing the chance of impurities and over passivization.^46^ The acetonitrile system is shelf stable for long periods.^43^ Acetonitrile as a solvent also has many desirable properties for high speed manufacturing such as low boiling point, low viscosity^47^ and low surface tension^48^ relative to the commonly used DMF,^49,50^ DMSO,^48,51^ and NMP^52,53^ systems as seen in Table 1. This is also seen in Fig. 1, which shows the low flow boundary limits for a 5 μm wet film (∼400 nm dry film for a 1 M solution). With an easily accessible gap height of 100 μm, this shows that acetonitrile has a maximum possible coating speed of 111 m min^−1^ relative to other common solvents (DMF = 58, NMP = 33, and DMSO = 29), neglecting changes to rheology due to additives. Furthermore, a low viscosity also allows higher coating speeds in relation to the so-called “critical” capillary number, where defects occur due to dynamic wetting failure at the upstream meniscus of the coating bead.^39^ Therefore, the acetonitrile perovskite system is better suited for fast manufacturing than traditional solvent systems as quantified in Fig. 1.

**Table 1:** Properties of solvents used for perovskite inks

Solvent	Boiling point (°C)	Viscosity (mPa s)	Surface tension (m Nm^−1^)
Acetonitrile	82	0.34	28.4
DMF	152	0.81	35.2
DMSO	189	1.99	42.8
NMP	202	1.66	40.8

However, many of the initial reports of the acetonitrile system require a two-step process, where the perovskite is deposited from acetonitrile, thermally annealed and later modified, usually with methylammonium chloride.^41,42^ This process has been described as work function shifting and surface passivating, thereby increasing radiative lifetimes which contribute to higher efficiency.^54^ Both processes require long annealing times for high efficiency and the multiple steps makes this process undesirable from a manufacturing standpoint.

The use of acid modifiers has been well studied in DMF based systems to control grain growth, morphology and electronic properties, but is currently unstudied in acetonitrile mixed solvent systems. In this study we explore the acid doping of acetonitrile based perovskites and apply it to the slot die coating process at room temperature in order to create a single step deposition method without the need of additional modification. We also observe drying defects in this system, and employ a novel method to control drying which shows a potential pathway for creating uniform large area films at room temperature. In addition, we show that short annealing times are possible with this system, demonstrating the applicability of this solvent system for the use in high speed manufacturing.

## 2 Results and discussion

**Fig. 2:**
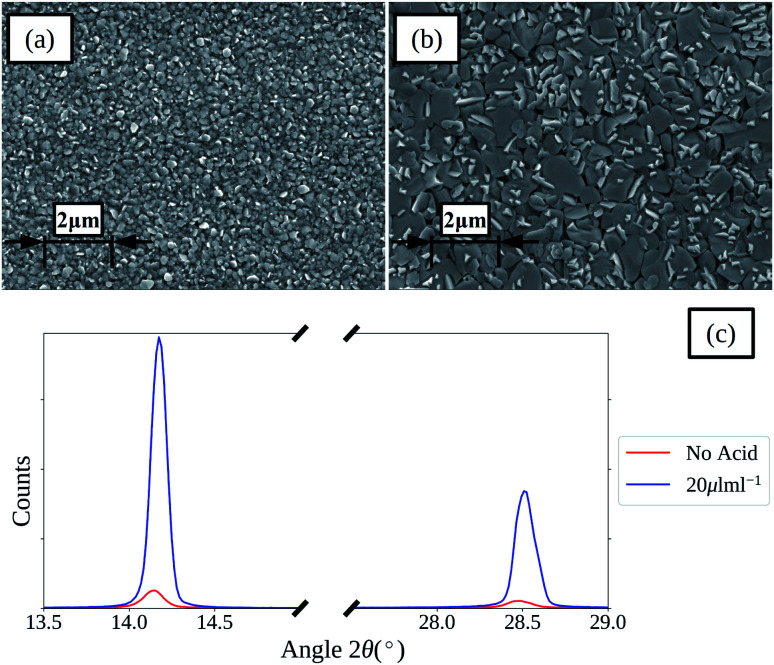
SEM images of spin coated perovskite films formed from ACN solvent based inks with no HCl_(aq)_ addition (a) and 20 μL mL^−1^ (b) (inset scale-bars represent 2 μm), and the XRD spectra (c).

An SEM image of a perovskite film deposited from a 1 M ACN solvent based ink using spin coating (film thickness of approx. 400 nm) is shown in Fig. 2a. The film is composed of many small grains with low preferential orientation, which is further shown by the XRD spectra given in Fig. 2c with relatively low intensities of the main 110 perovskite reflection with a peak centred around 2*θ* 14°.

**Fig. 3:**
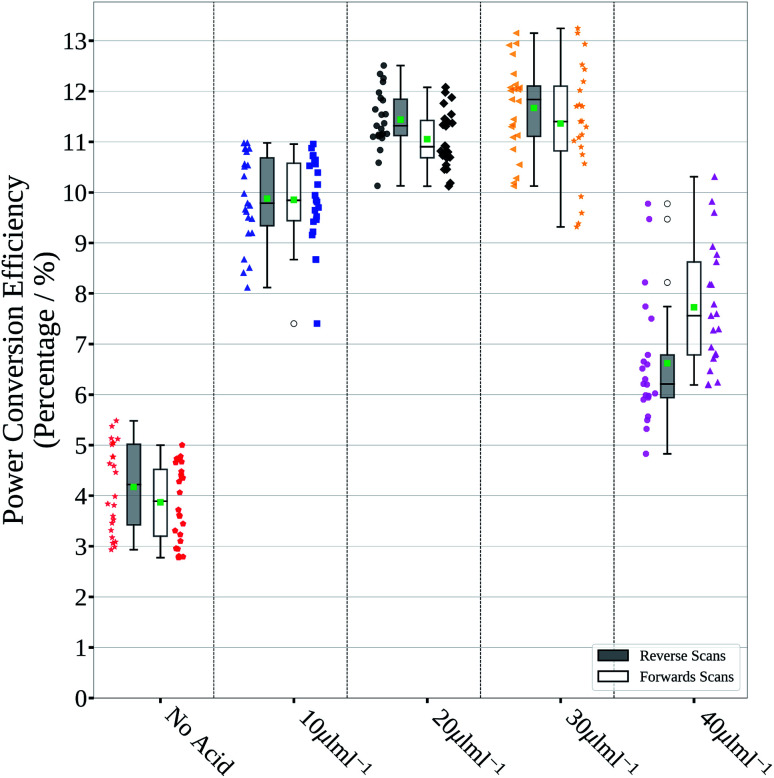
Power conversion efficiency of photovoltaic devices prepared from inks with various acid addition levels. The boxes represent the first and third quartiles, the horizontal black line the median, the upper whisker the data within 1.5 times the inter quartile range of the upper quartile and the lower whisker 1.5 times the inter quartile range of the lower quartile, the green square the mean and the open black dots outliers. The coloured full markers represent the individual scan results for the adjacent scan direction and corresponding split.

Devices with a p–i–n structure (stack details and fabrication methods are given in the Experimental methods section of the ESI†) made using these films result in poor *JV* scan photovoltaic parameters, as shown in Fig. 3 and S1 and summarized in Table S1,† with a median PCE of around 4%, mainly due to poor *J*_sc_ (short circuit current density). 1 M perovskite ink was used for spin coating to keep the perovskite ink concentration and film thickness used for slot-die coated films consistent. Spin coated 1.5 M ink was found to give thicker films (approx. 600 nm) and improved performance, but this was not suitable for slot-die coating as discussed below.

The addition of HCl_(aq)_ to conventional CH_3_NH_3_PbI_3_ precursor solutions has been shown to improve device performance.^55^ The addition of HCl_(aq)_ to the perovskite ink results in chloride addition to the perovskite film. This has been shown to have multifaceted roles that can improve device performance, through changes to crystal growth and the resulting film morphology and to the opto-electronic properties of the perovskite film, passivisation of trap states and improvements to interfaces between the perovskite and charge extraction layers.^56–62^

To improve the grain size and orientation of the perovskite films the use of hydrochloric acid as a chloride containing additive was investigated. SEM images and XRD spectra of perovskite films formed from ACN based inks with no acid addition and 20 μL mL^−1^ of HCl_(aq)_ are compared in Fig. 2a–c. The SEM images show that there is a clear increase in perovskite grain size and preferential orientation with the addition of HCl_(aq)_, which is further evidenced by the large relative increase in the intensity of the main perovskite 110 and 220 reflection peaks, at approx. 2*θ* 14.1 and 28.5°, shown in the XRD spectra. SEM images and XRD spectra of films produced with various levels of acid addition are shown in Fig. S2 and S3.† Films with greater HCl_(aq)_ addition levels show more distinct grain boundaries and for the greatest addition levels this results in large voids forming between the larger grains of the films, resulting in a drop in the relative intensity of the perovskite peaks shown in the XRD spectra for these films. Clearly a balance between improving the grain size, reducing the number of grain boundaries and avoiding large voids between grains exists.

**Fig. 4:**
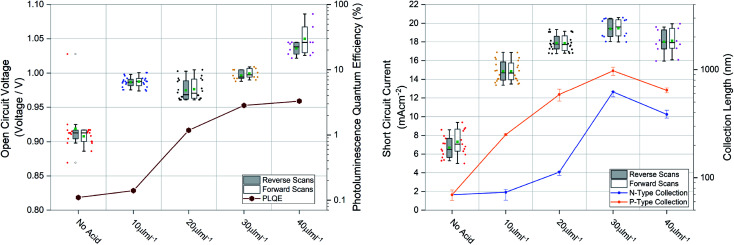
Open circuit voltage compared to photoluminescence quantum efficiency (PLQE) and short circuit current compared to n-type collection length and p-type collection length calculated from time resolved photoluminescence quenching measurements. The boxes represent the first and third quartiles, the horizontal black line the median, the upper whisker the data within 1.5 times the inter quartile range of the upper quartile and the lower whisker 1.5 times the inter quartile range of the lower quartile, green square the mean and the open black dots outliers. The coloured full markers represent the individual scan results for the adjacent scan direction and corresponding split.

Large grains and fewer grain boundaries would be expected to improve device performance by improving charge transport and reducing recombination at grain boundaries (thereby increasing open circuit voltage) whereas large voids between grains would result in shunt leakages in a full device stack manifesting in reduced fill factor. To confirm this we measured the steady state external photoluminescence quantum efficiency (PLQE), a measurement directly related to the *V*_oc_. We also employed a modified procedure of a previously reported technique^63,64^ to measure the charge collection length to each transport layer, directly related to the *J*_sc_. Both measurements plotted with their respective device performance statistics are shown in Fig. 4, the devices were prepared using 1.5 M ink, optimised for spin coating (approx. 600 nm film thickness). It was found that for optimized spin coating thicknesses, the PLQE increases with increasing acid additions as well as charge collection length. Although the PLQE rises drastically at 20 μL mL^−1^, the effects in the *V*_oc_ don’t manifest until higher additions. This indicates that although non-radiative recombination is lowered in the bulk initially, *V*_oc_ is still largely being driven by surface defects manifesting at the p–i and i–n interfaces. The collection length for both holes and electrons as well as the *J*_sc_ steadily rise with increasing acid additions but appear to be driven by hole collection. With higher additions there is a drop-off in collection length and therefore a slightly lower *J*_sc_. The results for spin coated films using the 1 M inks, seen in Fig. S4†, show similar behavior.

These effects manifest themselves with higher overall performance shown by the *JV* scan photovoltaic parameters found for devices made using these films, shown in Fig. 3 and S1,† and summarised in Table S1,† with lower performance for no HCl_(aq)_ addition and the lowest addition level, due to the smaller grain size. Higher performance was found for devices with 20 and 30 μL mL^−1^ HCl_(aq)_ addition, with a good balance between grain size and number of pin holes, and poor performance again for the devices made with the highest HCl_(aq)_ addition level (40 μL mL^−1^), which suffered from large shunt leakage due to the voids between the grains.

Generally for device performance and film stability to be maximized, chloride must be evolved from the film as it dries and the amount of chloride reduced substantially to a low level compared to that of iodide. Some studies have shown that small levels of residual chloride are beneficial for the perovskite optoelectronic properties and device performance^65^ and that chloride ions can migrate and concentrate preferentially at an interface.^66^ To determine if the surface of the films prepared with different levels of HCl_(aq)_ addition still contained chloride after the standard drying process (heating on a hot-plate at 100 °C for 60 minutes, optimised in the literature^41^), X-ray photoelectron spectroscopy (XPS) was used to analyse the chloride levels. Spectra showing the region of binding energies (204–194 eV) around the typical Cl 2p_3/2_ and Cl 2p_1/2_ peak positions are given in Fig. S5.† As expected for the film without acid addition, no peaks are found in this region and also for the films prepared from inks with 10 and 20 μL mL^−1^ HCl_(aq)_ addition no peaks are seen, but for films with 30, 40 and 50 μL mL^−1^ addition levels, peaks assigned to Cl 2p_3/2_ and Cl 2p_1/2_ are present. A summary of the elemental analysis is given in Table S2.† This indicates that for the higher addition levels not all chloride has been lost (from the surface of the films) during the drying process. Films prepared from inks with HCl_(aq)_ addition also display a shift in the position of the valence band edge, shown in Fig. S6.† This phenomenon has been reported for films prepared from ACN based solvent systems between films with and without a post treatment of MACl and has been related to improved electronic properties of the films for use in perovskite solar cells,^54^ and similar features are displayed here for films prepared using a single step process.

To be relevant for a manufacturing process with high throughput, short drying times are required and low drying temperatures are preferable. The device results shown so far have used a 60 minute at 100 °C drying stage for the perovskite layer, which is clearly too long to be applicable to a continuous roll-to-roll process operated at high line speeds as it would necessitate very long path lengths in drying ovens. ACN, the primary solvent in the formulations, has a low boiling point and is highly volatile, which suggests that solvent loss from films should happen rapidly at relatively low temperatures. The loss of solvent and the temperatures this occurs at was determined using thermogravimetric analysis (TGA), as shown in Fig. S7.† The mass loss of solvent occurs at under 100 °C with no other losses until the decomposition of solids at greater than 220 °C, and the perovskite solids content of the slot-die coating ink was approximately 40 wt%.

Having established that solvent can be rapidly lost from the films at low temperatures, reductions in the drying time were investigated. The standard method reported in the literature for the drying of perovskite films prepared from ACN based inks is 60 minutes at 100 °C on a hot-plate.^41^ Using this drying temperature and method for perovskite films prepared using the ink with 20 μL mL^−1^ HCl_(aq)_ addition, simply reducing the drying time to 2 minutes resulted in poor device performance, as shown in Fig. S8 and Table S3,† with a median forward scan PCE of 7.2% compared to 12.8% for films with a 60 minute drying time. The film with only 2 minutes drying also showed worse stability to humidity compared to the longer drying time, as shown in images of the films exposed to a humid atmosphere for a short time (Fig. S9†). The films with only 2 minutes drying gradually form large transparent crystallites across the surface, whereas the films with drying times of 60 minutes remain unchanged.

The changes in the films with drying times of 2, 10 and 60 minutes were further studied using XRD and XPS. The XRD spectra, shown in Fig. S10 and S11,† show that there is a shift in the pairs of main perovskite reflections, with peaks near 2*θ* 14 and 28°, from lower angles (approx. 13.9 and 28.2) to higher angles (approx. 14.1 and 28.5). The XPS spectra (Fig. S12†) show that with longer drying times there is a reduction in the intensity of peaks in the region of binding energies (204–194 eV), assigned to Cl 2p_3/2_ and Cl 2p_1/2_, showing that chloride is still present in the surface of the films with shorter drying times but is reduced and eventually absent with extended drying time. There is also a change in the region of binding energies, between 148 to 132 eV, near the peaks assigned to Pb^2+^ 4f_5/2_ and 4f_7/2_ (Fig. S13†), with an increase in the intensity of peaks with longer drying times, as well as an increase in the intensity of peaks near 141.8 eV and 136.8 eV that correspond to Pb^0^ 4f_5/2_ and 4f_7/2_. The values of these peaks are compared to those of other elements in the spectra and are summarised in Table S4,† which suggests that with extended drying time there is the formation of lead iodide and Pb^0^ on the surface of the films.^67^

This is further evidenced by the changes in the film morphology seen in SEM images (Fig. S14†) that show an increase in lighter shaded tetragonal rod shaped features that appear to be lead iodide crystallites on the surface of the perovskite film. The SEM images also show a coalescing of grains with greater drying time and a compacting of the films.

The results of drying the films for different times suggest that several things need to happen to result in films that will give good device performance. Namely, the evolution of and reduction in the level of chloride in the surface of the films, crystallisation and compaction of films, that would be expected to be beneficial to device performance. However, with an extended drying time the formation of lead iodide and Pb^0^ on the film surface can occur, which would be expected to be detrimental to device performance.

**Fig. 5:**
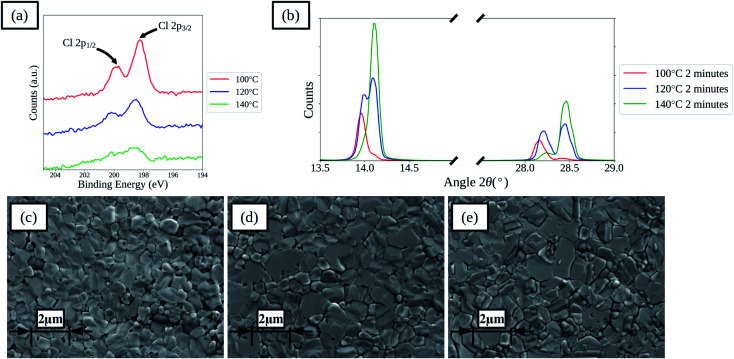
XPS spectra of perovskite films prepared from ACN solvent based inks with 20 μL mL^−1^ HCl_(aq)_ dried for 2 minutes at various temperatures (a), XRD spectra (b) and SEM images of films dried at 100 °C (c), 120 °C (d), and 140 °C (e) (inset scale-bars represent 2 μm).

Increasing the drying temperature was investigated as a method to reduce the time required to dry the films. Temperatures of 120 and 140 °C were used as these are compatible with the commonly used flexible substrate polyethylene terephthalate (PET) that has a maximum processing temperature of around 140–150 °C. Films and devices with drying times of 2, 10 and 60 minutes at 120 and 140 °C were prepared. For films with 2 minutes drying time XPS spectra show that there is a decrease in surface chloride levels for the films dried at higher temperatures, as shown in Fig. 5a, indicating that increasing the drying temperature is effective at removing chloride from the films more rapidly. XPS elemental analysis results for the films are further summarised in Table S4,† showing that with increased drying temperature the rate of loss of chloride from the film surface is increased for all times. XRD spectra of the films show that the rate of shift in the main perovskite peaks is increased, as shown in Fig. 5b for films dried for 2 minutes. SEM images, shown in Fig. 5c–e and S14,† of the films show that the higher temperature dried films become compact more rapidly and that the lighter coloured formations on the surface of the films form more rapidly. Comparing the films dried for 60 minutes between the three different temperatures shows a greater formation of the lighter coloured features on the films surface with higher temperatures, which correlates with a reduced ratio between the iodide and lead(ii) levels found in the surface of the films by XPS, suggesting the formation of lead iodide. In terms of device performance there are changes in both the levels of hysteresis between the forward and reverse current–voltage scan directions and in the relative performance of films prepared using the different drying regimes.

Fig. S16† summarizes the average of forward and reverse scan median PCEs and compares these to the chloride levels found at the surface of the films and the ratio of iodide to lead(ii) at the surface. Some general trends can be seen; when comparing films dried for 2 minutes there is an increase in performance with a reduction in chloride level, as also shown by films dried at 100 °C for longer times. When comparing performance of devices with 60 minutes drying, there is a decrease in performance for the highest temperature that correlates with a decrease in the iodide to lead(ii) ratio. The highest performance is found for devices with 10 minutes drying at 120 °C, where there is still some chloride present in the surface of the films (approx. 0.6%) and the ratio of iodide to lead is close to 3 : 1, as would be found in a stoichiometric perovskite. These devices also show the lowest level of hysteresis from looking at the ratio of median forward and reverse scan direction PCEs, suggesting higher quality films.

Clearly the presence of chloride in the surface of the film suggests that eliminating all chloride from the films might not be essential to achieving the highest performance, with the highest efficiencies achieved for devices with 0.6 and 0.8% surface chloride which is similar to the 1% reported in the literature for high performance devices, but the general trends suggest that high surface chloride levels (greater than 1–2%) can be used as an indicator of films that have not been dried sufficiently. The iodide to lead(ii) levels also suggest that this can be used as an indicator of films that have been over dried and have begun to degrade. The optimal drying conditions were also found to depend on the dry film thickness of the perovskite layer, with thicker films requiring longer drying times than those found for the films shown here, with dry film thickness of 350–400 nm. For instance a film of approximately 650 nm, with only 2 minutes drying at 140 °C was not stable towards humidity and resulted in poor performance. This can be rationalised by understanding that the evolution of chloride from the films will depend on the film thickness. Clearly the drying regime needs to be optimised for the particular film thickness used.

Having established a processing window for more rapid drying of the perovskite films, the transition to a potentially large area high throughput coating method was investigated using slot-die coating. The viscosity and surface tension of the ACN based perovskite ink was measured and is summarised in Table S5.† Given the solids content of the ink, a target 5 μm wet film thickness was selected to give an appropriate dry film thickness of between 350–450 nm. Considering the rheology of the ink, coating conditions of a speed of 1 m min^−1^ were selected and the use of a 1000 μm length meniscus guide, with an offset from the substrate (set from the lower edge of the meniscus guide) of approximately 150–250 μm was used. These conditions resulted in stable wet film formation of the coating, without entering a low-flow regime or causing flooding and loss of pre-metering and patterning.

Coatings were made using the ink with the addition of 20 μL mL^−1^ HCl_(aq)_, and rapid evaporation of solvent from the wet film caused an uneven moving solvent front pattern across the width of the dry film. At the edges of the solvent front the films have many large voids and pin-holes, which would result in shunt leakages in devices and lower performance, and in the areas between the solvent fronts the film formation is more uniform with fewer pin-holes and a continuous film.

**Fig. 6:**
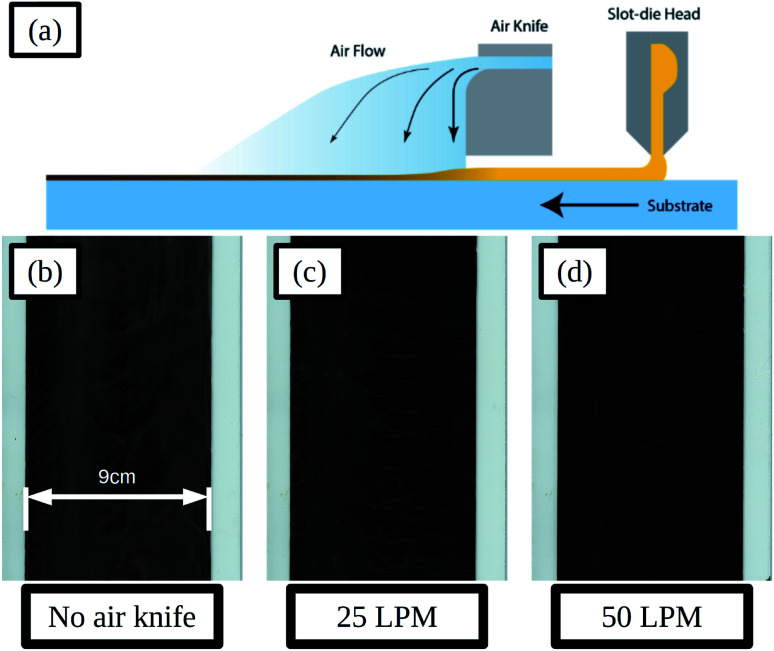
Schematic of slot-die coating and positioning of the air knife downstream of the coating head and air flow over the ink wet film (a), scans of slot-die coated perovskite films deposited using different air-knife settings of no air flow (b), 25 LPM (c) and 50 LPM (d), the inset scale-bar represents 9 cm.

To control the evaporation of solvent from the wet films and improve the uniformity of the dry film the application of a gas jet *via* an air-knife was used, and a schematic illustration of the slot-die coating process and air knife is given in Fig. 6a. The air-knife emits a high velocity stream of gas and entrains a flow of air from the area surrounding the gas stream. By directing the air-knife gas jet across the wet film, just after coating, the evaporation of solvent could be made more uniform and the rate increased.

Adjustments to the gas jet flow rate were made and the resulting dry films are shown in Fig. 6b–d. With a flow rate of 25 LPM (litres per minute) the film formation is improved compared to that without air-knife, with a more uniform surface and smaller areas between solvent fronts. SEM images of the films in the areas between solvent fronts and at the solvent front are compared in Fig. S17a and d and Fig. S17b and e† for no air knife and 25 LPM respectively. The areas between solvent fronts are similar in both cases and for the areas at the solvent front the 25 LPM film shows smaller pin-holes and voids compared to that without air-knife. Increasing the flow-rate further to 50 LPM resulted in further improvements to the film formation and much more uniform films with much smaller solvent fronts and areas between these, as shown Fig. 6d, and there was very little difference in film morphology in these areas seen in SEM images, shown in Fig. S17c and f.†

Devices, using ITO coated glass substrates with a sheet resistance of 15 Ω sq^−1^, were prepared using air-knife settings of 0, 25 and 50 LPM flow rates, and the *JV* curve photovoltaic parameters of these are summarised in Fig. S18 and Table S6.† With increased flow rate the performance increases across all parameters, as would be expected with the improvement in film formation. The optimised 50 LPM setting resulted in devices with a median forward scan PCE of 14.7%. This result demonstrates the potential to produce high performance perovskite solar cells using an industrially relevant coating method, through optimisation of the ink formulation and coating conditions.

To further demonstrate the potential of this method, devices were prepared using flexible substrates, which would be compatible with a roll-to-roll coating process. ITO coated PET was used as substrate, and the *JV* curve photovoltaic parameters of devices prepared on 50 Ω sq^−1^ ITO from either spin or slot-die coating are summarised in Table 2 and Fig. S19.† The devices prepared using slot-die coating show a slight improvement in performance, mainly due to a small increase in *V*_oc_, which could be due to the reduced handling required to produce the slot-die coated devices and the difficultly in spin-coating on flexible substrates. Devices were also prepared by slot-die coating on 15 Ω sq^−1^ ITO coated PET, these resulted in performance similar to that of devices produced on glass substrates, with a median forward scan PCE of 14%.

**Table 2:** Median *JV* scan photovoltaic parameters of spin coated and slot-die coated perovskite devices prepared on flexible PET substrates with different sheet resistances of ITO

Coating method	ITO sheet resistance (Ω sq^−1^)	Scan direction	*V*_oc_ (V)	*J*_sc_ (mA cm^−2^)	FF (%)	PCE (%)
Spin	50	Forward	0.94	18.7	73	12.1
		Reverse	0.93	18.6	55	9.0
Slot-die	50	Forward	0.98	18.5	72	12.8
		Reverse	0.99	18.7	64	11.8
Slot-die	15	Forward	0.99	19.9	71	14.0
		Reverse	0.98	20.1	59	11.6

**Fig. 7:**
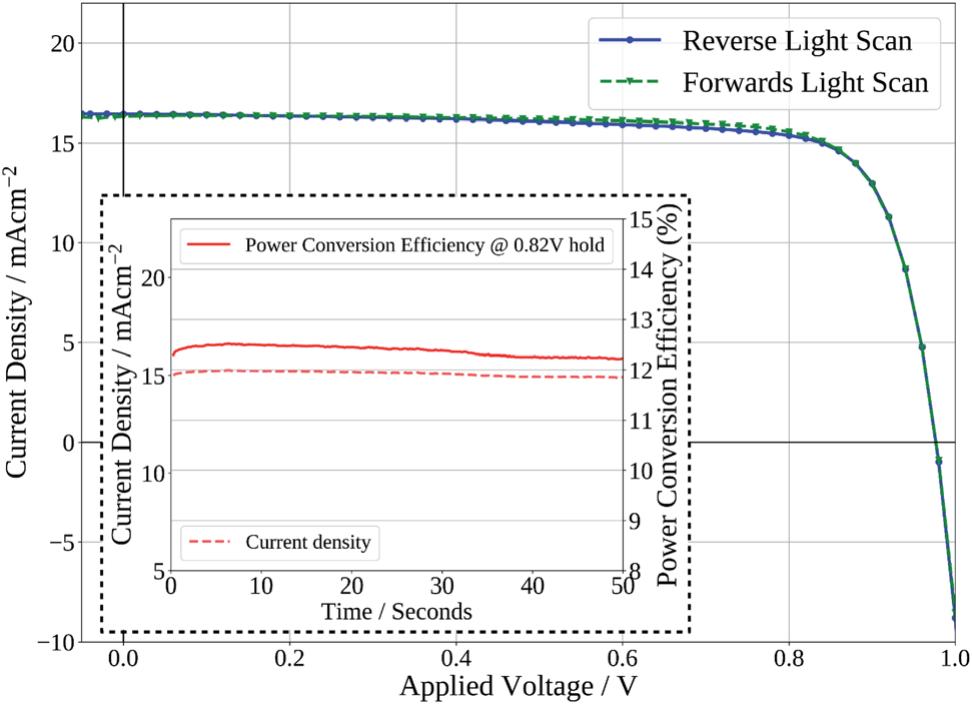
Current density–voltage curve of device produced with slot-die coated perovskite film dried for 2 minutes on flexible PET substrate. Inset – stabilised power output of device held at maximum power point voltage.

Finally, Fig. 7 shows the stabilised power output of a flexible slot-die coated perovskite device on 15 Ω sq^−1^ ITO coated PET with a short 2 minute drying time of the perovskite layer, on a hot-plate at 140 °C. The device shows a stabilised PCE of 12.2%, further demonstrating the potential of the ink developed to be used to produce high efficiency devices from slot-die coated films with short drying times, on flexible substrates.

## 3 Conclusions

This work demonstrates the formulation of a perovskite ink developed for use with the industrially relevant coating method of slot-die. The use of an acetonitrile based solvent system and consideration of the ink rheology shows that the ink is well suited for slot-die coating at high line speeds. Through the use of a chloride containing additive in the ink the crystallisation of the perovskite film is improved, with improvements in the grainsize and reduction in the number of grain boundaries, resulting in improvements in device performance from both spin coated films and those produced by slot-die coating. The acetonitrile based ink rapidly dries when slot-die coated and an unstable solvent evaporation front forms defects in the perovskite film, therefore to control and improve the solvent drying profile the use of an air-knife is implemented, which produces homogeneous films with improved uniformity and fewer defects. To achieve optimal device performance it was found that the perovskite films needed to be dried sufficiently to reduce surface chloride levels to around 0.6–0.8% (a level concurrent with values reported in the literature), the films also compacted and grainsize increased with extended drying times or drying temperatures. If films were dried for too long at a particular temperature it was found that lead iodide and/or Pb^0^ would form on the surface of the layer and this was detrimental to device performance. By increasing the drying temperature to 140 °C, the maximum processing temperature suitable for a PET based substrate, a short drying time of 2 minutes could be used to produce devices on both rigid glass and flexible PET substrates that displayed high performance from slot-die coated perovskite films. The stabilised performance of 12.2% achieved improves on reports of devices produced with a similar P–I–N device stack and perovskite layer deposited from an ACN solvent ink, deposited by blade coating, not using chloride containing additives, that achieved a stabilised PCE of 10.5%,^26^ demonstrating the importance of the HCl_(aq)_ additive for achieving high performance. The use of ACN solvent system perovskite inks has been reported for slot-die coated devices on flexible glass and using an N–I–P device stack with SnO_2_ interlayer,^26^ again without the use of chloride containing additives, the device performance reported is higher than for the P–I–N device stack, and the results presented here suggest further gains in efficiency could be achieved through the choice of chloride containing additives in the perovskite ink.

## Conflicts of interest

There are no conflicts to declare.

## Supplementary Material

RA-009-C9RA06631D-s001
