# Supplementary material for: Acetonitrile based single step slot-die compatible perovskite ink for flexible photovoltaics
Source: RSC Adv. 2019 Nov 15;9(64):37415–23. doi: 10.1039/c9ra06631d (PMC9075525; doi:10.1039/c9ra06631d)
Supplement: RA-009-C9RA06631D-s001 [file RA-009-C9RA06631D-s001.pdf]

Electronic Supplementary Material (ESI) for RSC Advances.
This journal is © The Royal Society of Chemistry 2019

Electronic Supplementary Information

Acetonitrile Based Single Step Slot-Die Compatible Perovskite Ink For Flexible Photovoltaics

Daniel Burkitt[a], Richard Swartwout[b], James McGettrick[a], Peter Greenwood[a], David Beynon[a], Roberto Brenes[b], Vladimir Bulović[b], Trystan Watson[a]

[a] *SPECIFIC, College of Engineering, Swansea University, Bay Campus, SA1 8EN Swansea, UK. E-mail: daniel.burkitt@swansea.ac.uk*

[b] *Research Laboratory of Electronics, Massachusetts Institute of Technology, 77 Massachusetts Avenue, Cambridge, MA 02139, USA*

1 Experimental Methods

1.1 Ink Preparation.

Perovskite ink formulations were synthesized based on a modified recipe of a previous report[1]. Methylamine Solution (40% wt in H_20, 426466), Acetonitirle (Anhydrous 99.8%, 271004), and Hydrochloric Acid (37%, 320331) were all purchased from Sigma. Lead(II) Iodide (99.99%, L0279) was purchased from TCI America. Methylammonium Iodide (MS101000) was purchased from GreatCellSolar. Drierite non-indicating absorbent was purchased from VWR. In a typical synthesis, 10.675g of PbI_2, 3.472g of MAI, and 30mL of anhydrous acetonitrile were added to a 50mL Erlenmeyer flask. Solids were weighed under nitrogen and solvents were added to the flask from ambient conditions. A modified borosilicate glass tube, bent under a natural gas flame in order to avoid the sir bar was added into the perovskite flask, fitted with an ace-glass ground glass vacuum adapter. This allowed for partial control of the atmosphere within the flask. A fritted glass bubbler was then loaded with 100mL of methylamine solution. Methylamine solution was then bubbled under ambient conditions with dry nitrogen at 150ccm and passed through a 450g Drierite column and into the perovskite precursor solution. All connections were made with Tygon tubing. During bubbling the perovskite precursor solution was stirred at 800RPM with a 25mm, teflon coated stir bar. The solution would go from deep black, to dull grey, to a yellow particulate and finally a clear yellow solution over the course of about an hour. Once no particulates were seen in the solution, the bubbling rate was turned down to 50ccm and allowed to bubble for 2 minutes longer to avoid introducing excess MA gas. Once finished, the fabricated solution was transferred to a 40mL brown septum sealed vial, the cap was taped shut, and stored in lab refrigerator held at 4°C. The perovskite stock solution was used over the course of about 4 months. For slot die coated films, the stock solution was diluted with 1:2 anhydrous acetonitrile to bring the molarity to about 1M of total solute or 0.5M of perovskite. All solutions were treated with concentrated hydrochloric acid before use with the concentrations present in the main text. Particles would form directly after adding the HCL, but would quickly redissolve. The working time for all solutions at this point was around 1-2 hours.

1.2 Device Fabrication Methods.

A p-i-n device stack of glass substrate coated with indium tin oxide (ITO) transparent conducting electrode, poly(2,3-dihydrothieno-1,4-dioxin)-poly(styrenesulfonate) (PEDOT:PSS) hole transport material (HTM), methylammonium lead triioide perovskite active layer, [6,6]-phenyl-C_{61}-butyric acid methyl ester (PCBM) electron collection layer (ECL), bathcuproine (BCP) buffer layer and silver top electrode was used.

Spin coated devices were prepared on ITO coated glass substrates (15Ω/sq), cleaned sequentially by rinsing and ultrasonication with acetone, deionised water and 2-propanol. Substrates were further treated by a plasma cleaning process, using air as a feed gas. Spin coating was carried out in a humidity controlled environment (approximately 30% RH). PEDOT:PSS (Clevios P VP AI 4083 - Heraeus) was filtered using a 0.45μm PVDF syringe filter then (250μl) spin coated on the substrate at 1000RPM with acceleration of 1000RPMs^{-1} for 5s followed by 5000RPM for 10s, then moved to a hot-plate and dried for 10 minutes at 120°C. Once the substrates had cooled to room temperature perovskite ink (100μl) was spin coated at 2000RPM with an acceleration of 2000RPMs^{-1} for 60s, within the first 5s of the spin process the films turned a dark colour. Perovskite films were moved directly from the spin-coater to a hot-plate and dried at the temperatures and for the times given in the main text. Once the substrates cooled to room temperature PCBM ([60]PCBM Solenne 99.5% or Nano-C 99.9%) ink (40mg/ml in chlorobenzene) at 80°C (100μl) was spin coated on the films at 3000RPM with an acceleration of 3000RPMs^{-1} for 45s. Bathcuproine (Sigma) in anhydrous ethanol (sigma) (0.5mgml^{-1}) was spin coated (approx. 200μl) on the films at 7000RPM with an acceleration of 7000RPMs^{-1} for 10s. Finally silver top contacts (approximately 200-300nm thickness) were applied to the devices using thermal evaporation under vacuum, using shadow masks to define pixel areas.

Slot-die coated devices were prepared on pre-patterned ITO coated glass substrates (15Ω/sq) (10cm by 20cm) or ITO coated PET substrates (15 or 50Ω/sq). The slot-die head gap height was set using micrometer adjustment screws and feeler gauges. An air knife (Exair Standard Air Knife) was positioned at the downstream side of the coating head and compressed nitrogen used as the feed gas. Substrates were cleaned by rinsing with acetone, water and 2-propanol sequentially from wash bottles, followed by drying with compressed nitrogen. PEDOT:PSS (Clevios P VP AI 4083 - Heraeus) was diluted with 2-propanol (1:3vol/vol) then filtered using a 0.45μm PVDF syringe filter. The slot-die head was fitted with a 50μm thick shim and a meniscus guide with 1000μm tab length, coatings were made at a speed of 0.2mmin^{-1}, across a coating width of 90mm, with an ink flow rate of 0.18mlmin^{-1} to give an approximately 10μm wet film thickness. Films were dried on a hot-plate at 120°C for 10 minutes. For the perovskite coatings a slot-die head, again, fitted with 50μm thick shim and a meniscus guide with 1000μm tab length was used, with a 90mm coating width. A coating speed of 1mmin^{-1} was used, with an ink flow rate of 0.45mlmin^{-1}, to give an approximately 5μm wet film thickness. Films were dried on a hot-plate for the times and temperatures given in the main text. The substrates were then diced in to 28 by 28mm pieces and the remaining layers deposited by spin coating and thermal evaporation.

1.3 Optical Characterization Methods.

Samples were prepared by first spin coating hydroxypropyl cellulose (HPC) (10mg/mL in water) onto glass at 3000RPM for 1 minute with an acceleration of 3000RPMs^{-1}. The thin films of HPC were then annealed at 100C for 10 minutes on a hotplate. Perovskite was then spin coated onto the HPC and treated using the same conditions as found in the Device Fabrication Methods section. The perovskite was then coated with PMMA (10mg/mL in chlorobenzene), spin coated at 2000RPM for one minute with an acceleration of 2000RPMs^{-1}. For the semiconductor bi-layer structures needed for electron and hole collection modeling, HPC and PMMA were replaced with PEDOT:PSS or PCBM respectively.

Time correlated single photon counting fluorescence measurements (TCSPC) for time resolved photoluminescence (TRPL) were performed using a PicoQuant PDL 800-D driver, LDH series 405nm laser, MPD series avalanche photodiode and TimeHarp 260 timing electronics. Measurements were conducted at 1MHz and under low injection conditions. Charge collection lengths were modeled using a finite element technique assuming monomolecular (linear) decay and perfect interfacial quenching[2]. For low injection conditions where bi-molecular recombination is semi-linear, this gives a reasonable estimation of charge lifetime while allowing for ease of modeling. Film thickness was measured using optical ellipsometry. Error bars shown in the main script in Figure 1 and figure S4 are errors in fitting photoluminescence

lifetimes as well as fitting film thickness.

For steady state photoluminescence quantum yield (PLQE) measurements the same 800-D driver and LDH series 405nm laser were used in conjunction with a 4 inch integrating sphere and an Ocean Optics USB-4000 spectrometer. PLQE was calculated using the three measurement technique[3].

1.4 Other Characterization Methods

1.4.1 Scanning Electron Microscopy

Scanning Electron Microscope (SEM) images were taken using a Jeol JSM-7800F field emission gun electron microscope, adjusting the working distance around 10mm and using an acceleration voltages of 5kV and a probe current of the order of 9μA.

1.4.2 X-ray Diffraction

X-ray diffraction (XRD) measurements were taken using a Bruker D8 Discover instrument with a CuKα beam at 40 kV and 40 mA.

1.4.3 X-ray Photoelectron Spectroscopy

X-ray photoelectron spectroscopy (XPS) was collected on an Axis Supra (Kratos Analytical, Manchester UK) using a monochromated Al Kα X-ray source. In order to prevent differential charging, the integral Kratos charge neutraliser was used throughout.

All UHV processes were carried out in the dark to limit any Pb^0 evolution[4] and similarly N(1s) and Pb(4f) envelopes were collected first to limit any sample damage. Survey scans with a pass energy of 160 eV were used to identify the species present on the surface. High resolution scans were carried out at a pass energy of 40 eV, step size of 0.1 eV, with the dwell times and number of sweeps varied to improve signal.

Data analysis was carried out using CasaXPS (2.3.17dev6.4k) using the Kratos RSF library. Shirley backgrounds were used in all cases except N(1s), where the overlap of the broad Pb($4d_{5/2}$) line meant a U 2 Tougaard background represented the baseline more accurately. The default GL lineshape was used for synthetic components, and the binding energy scale was calibrated so that the CxHy component of the fitted C(1s) spectra is found at 284.8 eV. Peaks were identified with reference to the NIST XPS database[5] and Lindblad et al.[6].

1.4.4 Thermogravimetric Analysis

Thermogravimetric analysis (TGA) experiments were performed using a PerkinElmer STA6000. Samples of approximately 20mg were placed in a ceramic crucible within the instrument furnace. Thermal equilibrium between sample and crucible was ensured at the beginning of the experiment by a one-minute isothermal hold at 30°C. After this period the samples were heated to 800°C at 10°Cmin^{-1} whilst the sample weight was logged at 0.12 second intervals. A baseline experiment was performed under identical conditions using an empty crucible, these data were subtracted from the sample data to improve resolution by removing the inherent noise and curvature of the instrument baseline. The TGA data plots show the sample weight as a function of furnace temperature. Solids content (approximately 40 wt%) was determined at 100°C when all acetonitrile and water from added $HCl_{(aq)}$ had evaporated, this can be seen as a plateau in the weight curve, before the onset of the breakdown of the perovskite material.

1.4.5 Device Testing Methods

Current-voltage (IV) testing of devices was performed using a Keithley 2400 source measure unit and Oriel class AAA (Newport Oriel Sol3A) solar simulator as light source, calibrated to AM1.5 one sun equivalent intensity using a reference cell with a KG5 filter (Newport Oriel 91150-KG5). Cells were masked to $0.09cm^2$ for photovoltaic testing and current-voltage curves collected in reverse and forwards scan directions between 1.1 and -0.1V at a scan rate of approximately $0.15V^{-1}$, in two-wire mode, 5 seconds of light soaking at open circuit was applied to the cell before scanning. Stabilised power output measurements were collected holding the cell at maximum power point voltage (Vmp) and measuring the output current for 50s.

1.4.6 Rheological Measurements

Viscosity measurements were performed using a Malvern Bohlin Gemini 200 Nano HR rheometer, with a temperature controlled stage. Measurements were made across a range of shear rates, all inks were found to be Newtonian at the shear rates used, viscosity measurements are quoted as the value found for a shear rate of $2000s^{-1}$.

Contact angle and surface tension measurements were performed with a FTA32 system and for surface tension measurements a Pendant Drop Shape fitting routine used.

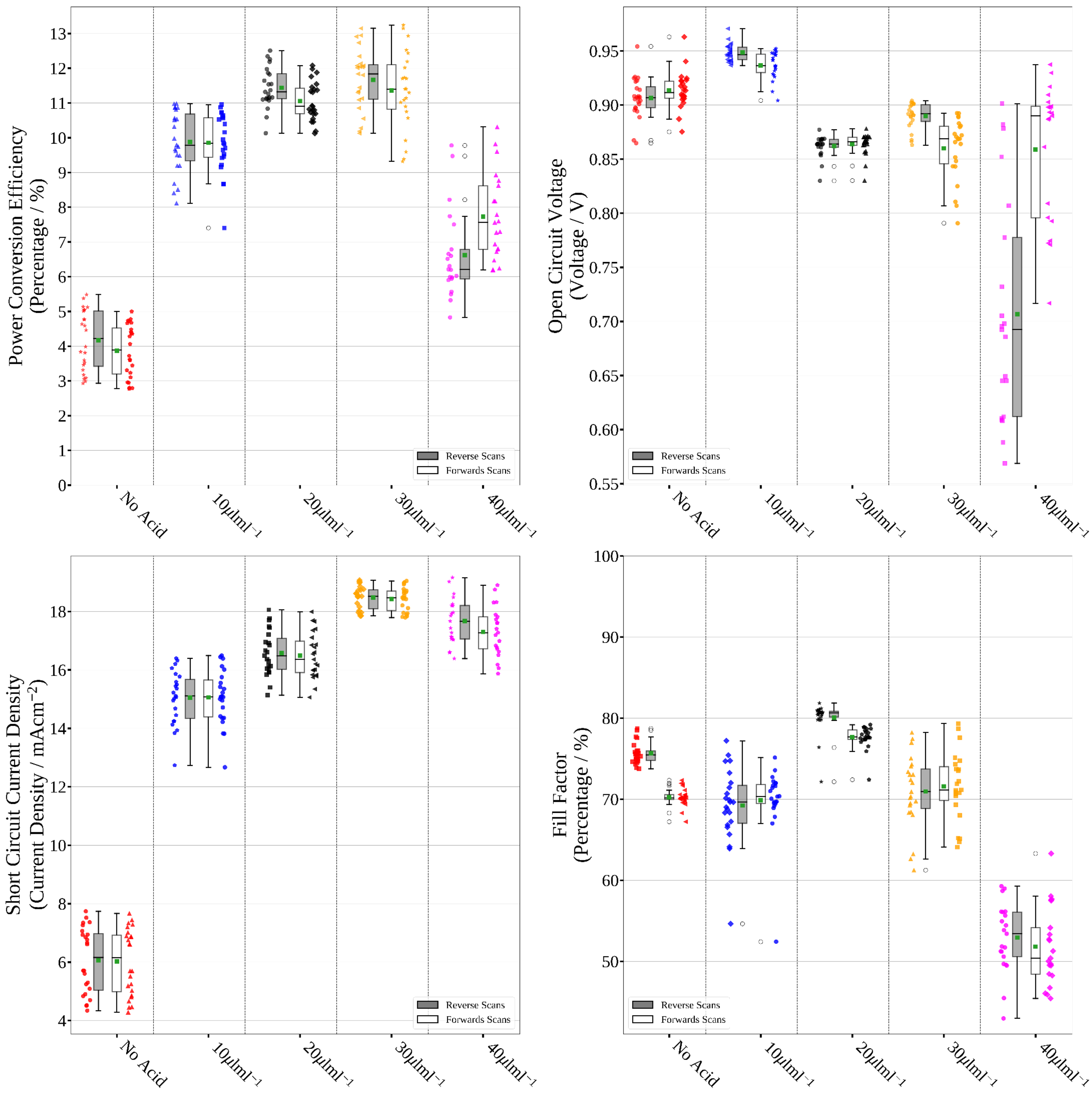

Figure S1: Box-plots of JV scan photovoltaic parameters for 0.09cm^2 cells with various acid addition levels to the perovskite ink. The boxes represent the first and third quartiles, the horizontal black line the median, the upper whisker the data within 1.5 times the inter quartile range of the upper quartile and the lower whisker 1.5 times the inter quartile range of the lower quartile, green square the mean and open black dots outliers. The coloured full markers represent the individual scan results for the adjacent scan direction and corresponding split.

Table S1: Median JV scan photovoltaic parameters of spin coated devices prepared using perovskite formulations with different $HCl_{(aq)}$ addition levels.

$HCl_{(aq)}$ ($\mu l ml^{-1}$)	Scan Direction	Voc (V)	Jsc ($mAcm^{-2}$)	FF (%)	PCE (%)
0	Forwards	0.91	6.2	70	3.9
	Reverse	0.91	6.2	75	4.2
10	Forwards	0.94	15.1	70	9.8
	Reverse	0.95	15.1	70	9.8
20	Forwards	0.87	16.4	78	10.9
	Reverse	0.86	16.5	81	11.3
30	Forwards	0.87	18.5	71	11.4
	Reverse	0.89	18.5	71	11.8
40	Forwards	0.89	17.3	50	7.6
	Reverse	0.69	17.7	53	6.2

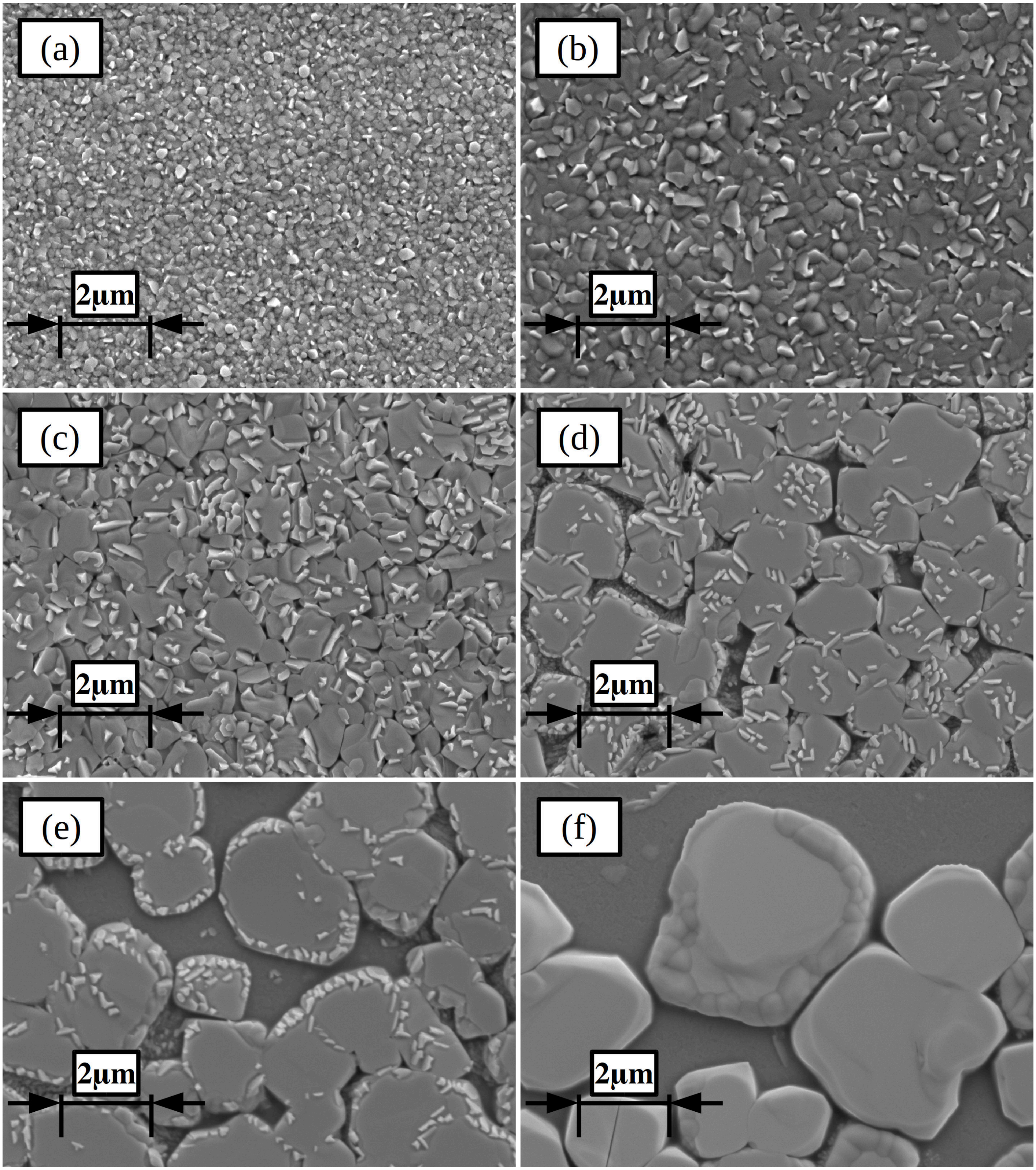

Figure S2: Top view SEM images of spin coated perovskite films on PEDOT coated ITO coated glass substrates with various hydrochloric acid addition levels. (a) No acid additon, (b) $10\mu lml^{-1}$, (c) $20\mu lml^{-1}$, (d) $30\mu lml^{-1}$, (e) $40\mu lml^{-1}$ and (f) $50\mu lml^{-1}$. Inset scale bars represent 2 microns.

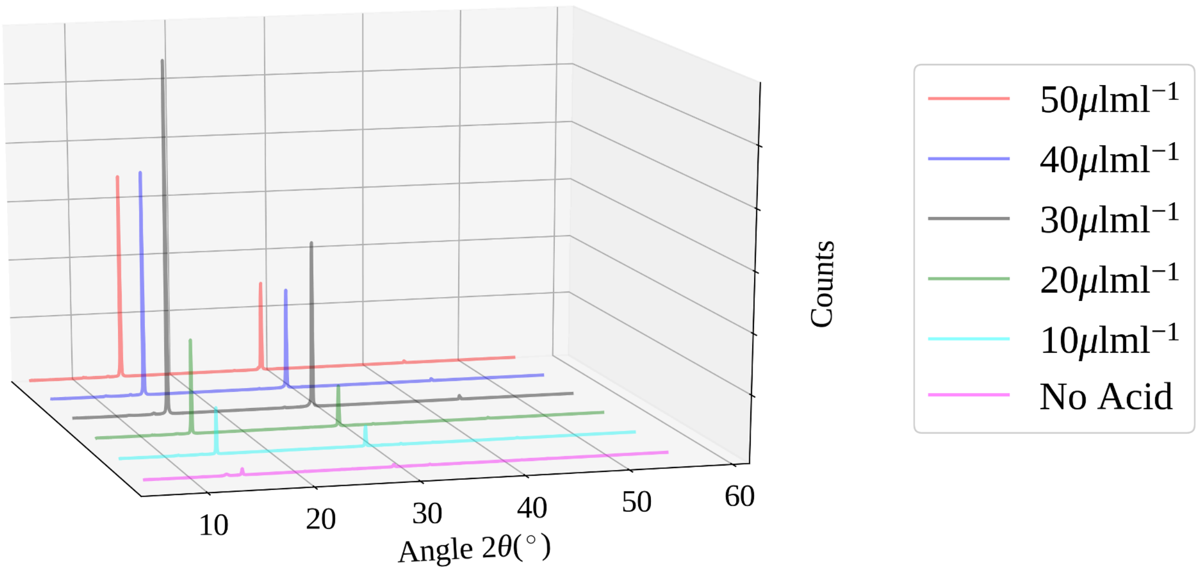

Figure S3: X-ray diffraction spectra of spin coated perovskite films on PEDOT coated ITO coated glass substrates with various hydrochloric acid addition levels, using 1M perovskite ink.

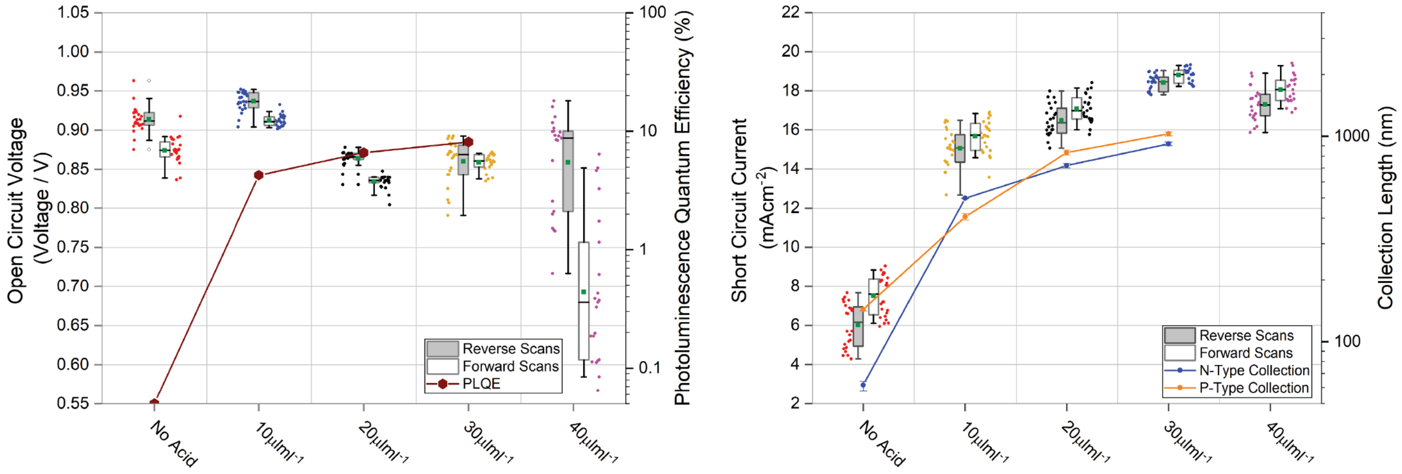

Figure S4: Photoluminescence quantum efficiency and device JV photovoltaic scan parameters with various hydrochloric acid addition levels, using 1M perovskite ink.

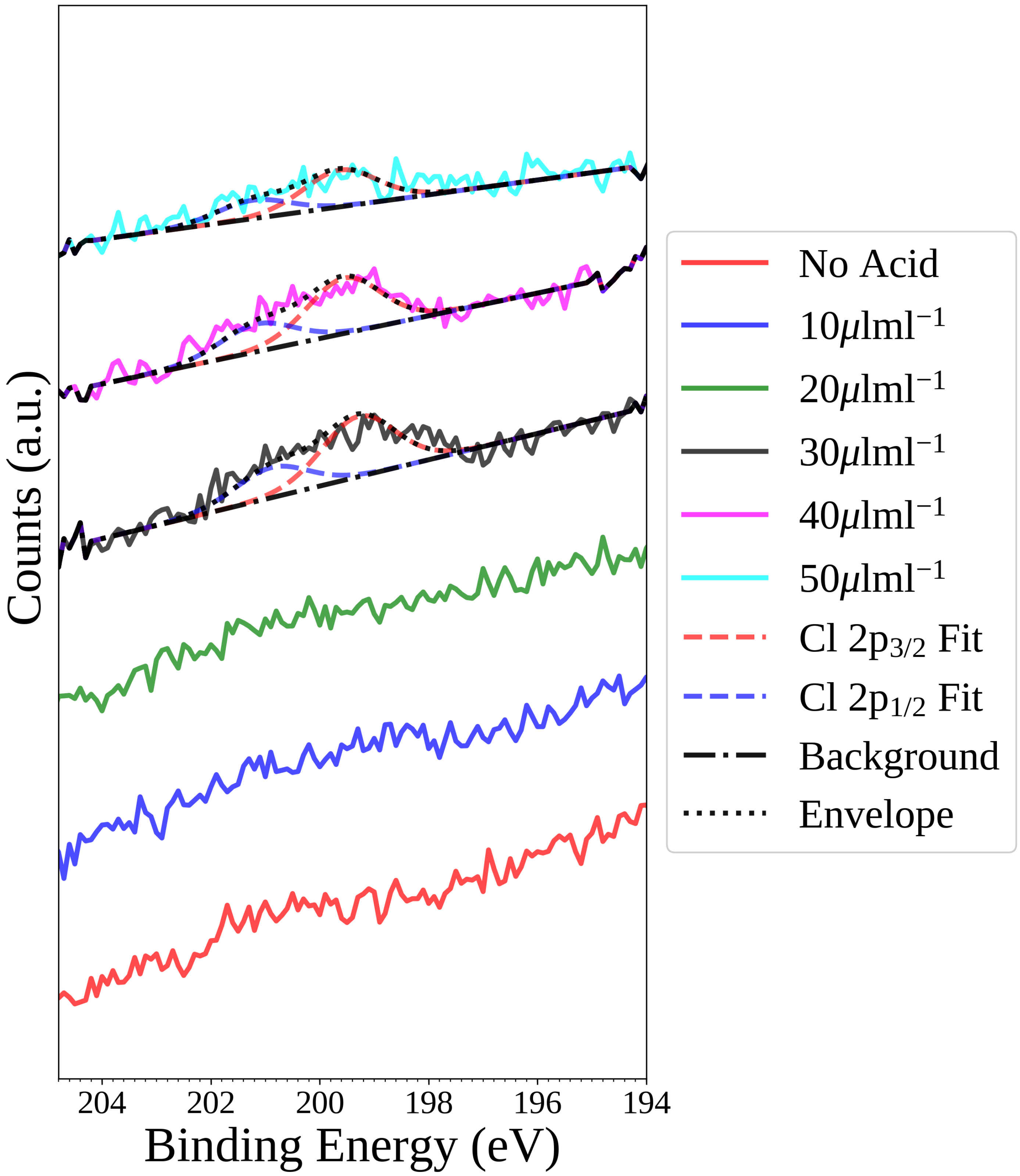

Figure S5: X-ray photoelectron spectroscopy spectra showing the change in the chloride $2p_{3/2}$ and $2p_{1/2}$ peaks for perovskite films made from inks with various $HCl_{(aq)}$ addition levels.

Table S2: X-ray photoelectron spectroscopy elemental distribution data for perovskite films prepared from formulations with various acid addition levels.

$HCl_{(aq)}$ (μlml^{-1})	Carbon	Chlorine	Iodine	Nitrogen	Oxygen	Lead(II)
0	27.52	0	41.04	4.83	4.41	21.33
10	31.13	0	38.22	6.70	6.06	17.05
20	34.07	0	36.55	6.37	6.17	16.28
30	38.41	0.38	30.53	5.67	8.99	14.62
40	41.50	0.33	27.45	7.10	10.41	12.03
50	46.60	0.19	24.22	9.94	9.92	7.65

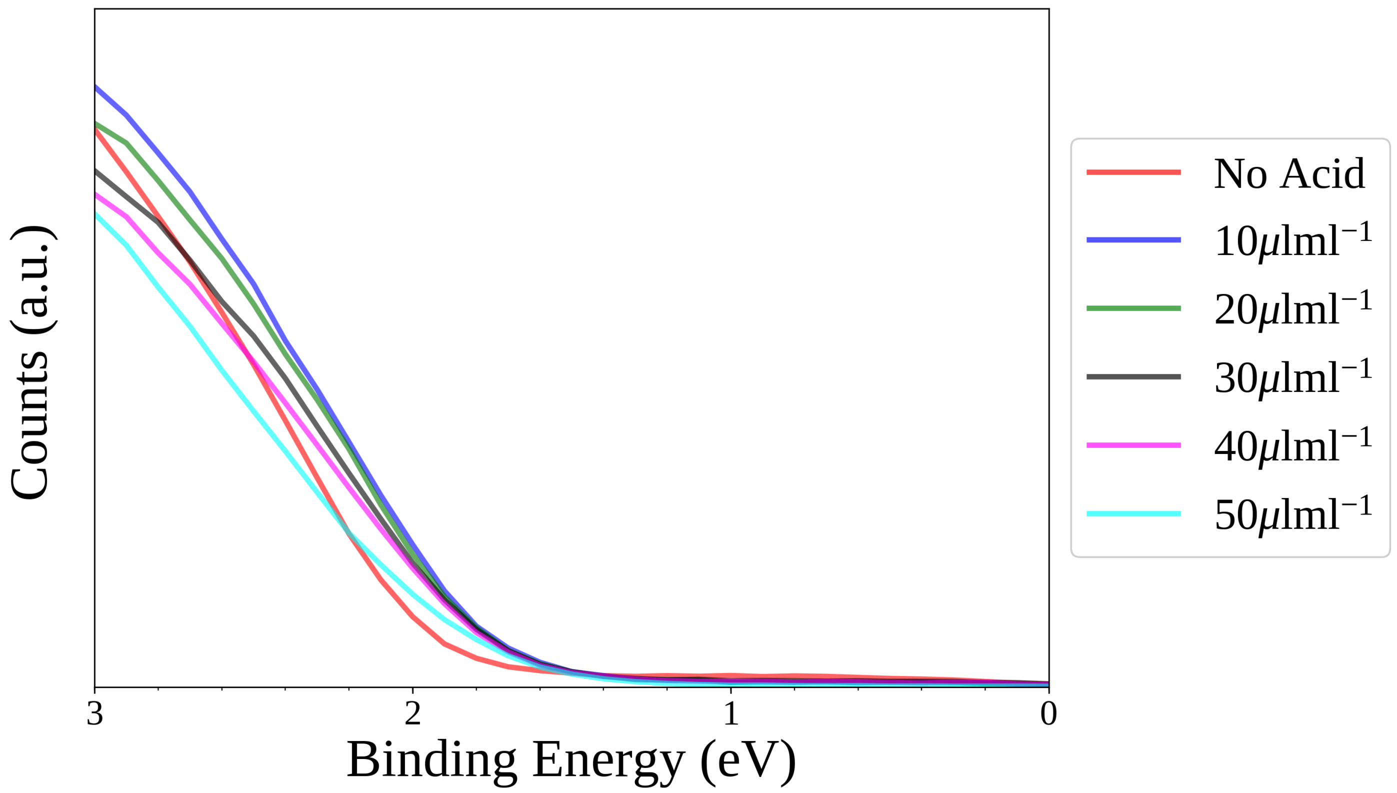

Figure S6: X-ray photoelectron spectroscopy spectra showing the change in the valance band levels for perovskite films made from inks with various $HCl_{(aq)}$ addition levels.

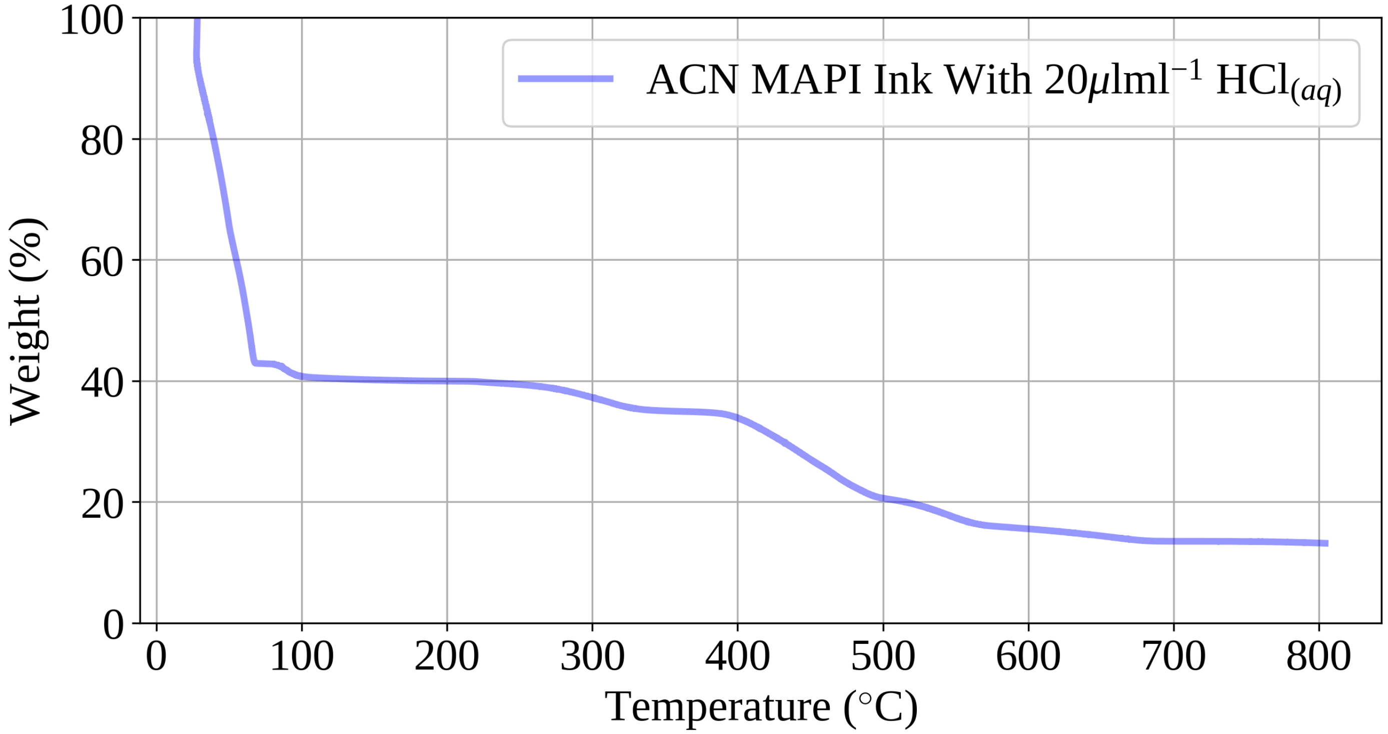

Figure S7: Thermogravimetric analysis of ACN perovskite ink formulation with 20μlml^{-1} HCl$_{(aq)}$ addition.

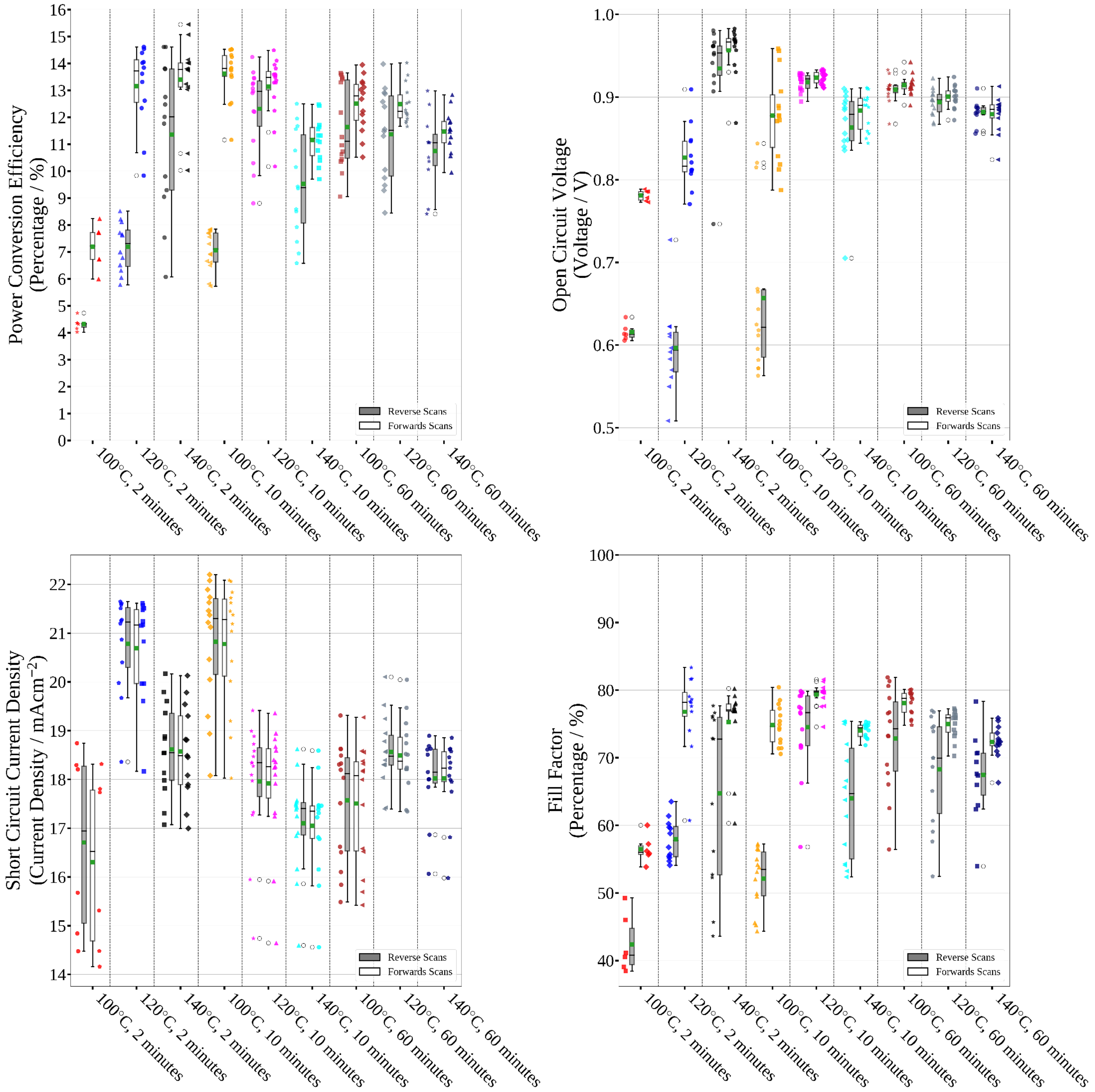

Figure S8: Box-plots of JV scan photovoltaic parameters for 0.09cm^2 cells with various drying temperatures and times of the perovskite films, prepared using ACN perovskite ink formulation with 20μlml^{-1} $HCl_{(aq)}$ addition. The boxes represent the first and third quartiles, the horizontal black line the median, the upper whisker the data within 1.5 times the inter quartile range of the upper quartile and the lower whisker 1.5 times the inter quartile range of the lower quartile, green square the mean and open black dots outliers. The coloured full markers represent the individual scan results for the adjacent scan direction and corresponding split.

Table S3: Median JV scan photovoltaic parameters of spin coated devices prepared using perovskite films with different drying times and temperatures, ACN perovskite ink formulation with $20\mu lml^{-1}$ $HCl_{(aq)}$ addition.

Temperature (°C)	Time (Minutes)	Scan Direction	Voc (V)	Jsc ($mAcm^{-2}$)	FF (%)	PCE (%)
100	2	Forwards	0.78	16.5	56	7.2
	2	Reverse	0.61	16.9	41	4.3
120	2	Forwards	0.82	21.2	78	13.7
	2	Reverse	0.59	21.2	58	7.3
140	2	Forwards	0.97	18.5	77	13.8
	2	Reverse	0.95	18.5	73	12.0
100	10	Forwards	0.88	21.3	75	13.8
	10	Reverse	0.62	21.3	53	7.1
120	10	Forwards	0.93	18.3	80	13.5
	10	Reverse	0.92	18.3	77	13.0
140	10	Forwards	0.89	17.3	74	11.2
	10	Reverse	0.88	17.4	65	9.4
100	60	Forwards	0.91	18.1	79	12.8
	60	Reverse	0.91	18.1	74	11.1
120	60	Forwards	0.90	18.4	76	12.2
	60	Reverse	0.90	18.5	70	11.5
140	60	Forwards	0.89	18.2	72	11.5
	60	Reverse	0.88	18.2	68	11.1

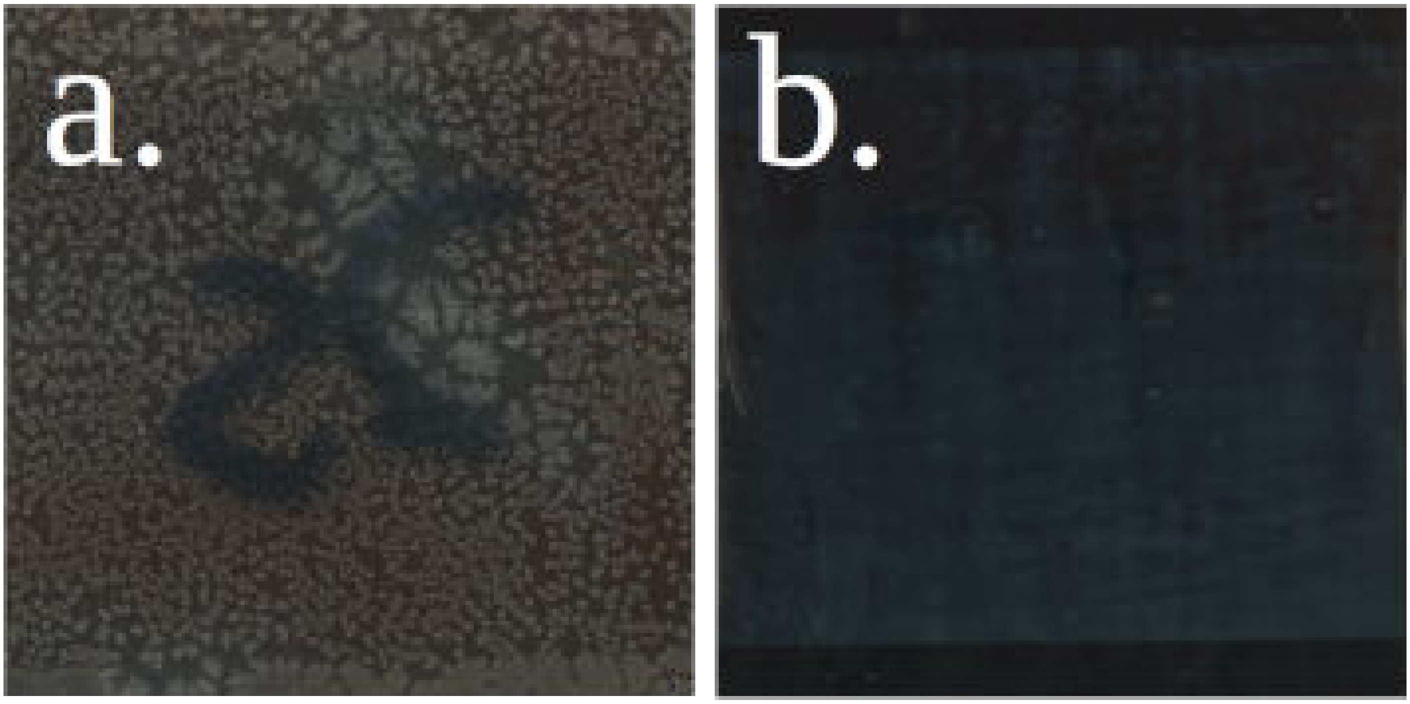

Figure S9: Photographs of perovskite films prepared using an ink with $HCl_{(aq)}$ addition level of $20\mu l ml^{-1}$ and dried on a hot plate at 100°C for either 2 (a.) or 60 (b.) minutes then exposed to humid air (50-60% relative humidity) for several hours. The film with only 2 minutes drying shows recrystallisation of the film to form large transparent crystallites and allows the writting on the reverse of the substrate to be visible through the film.

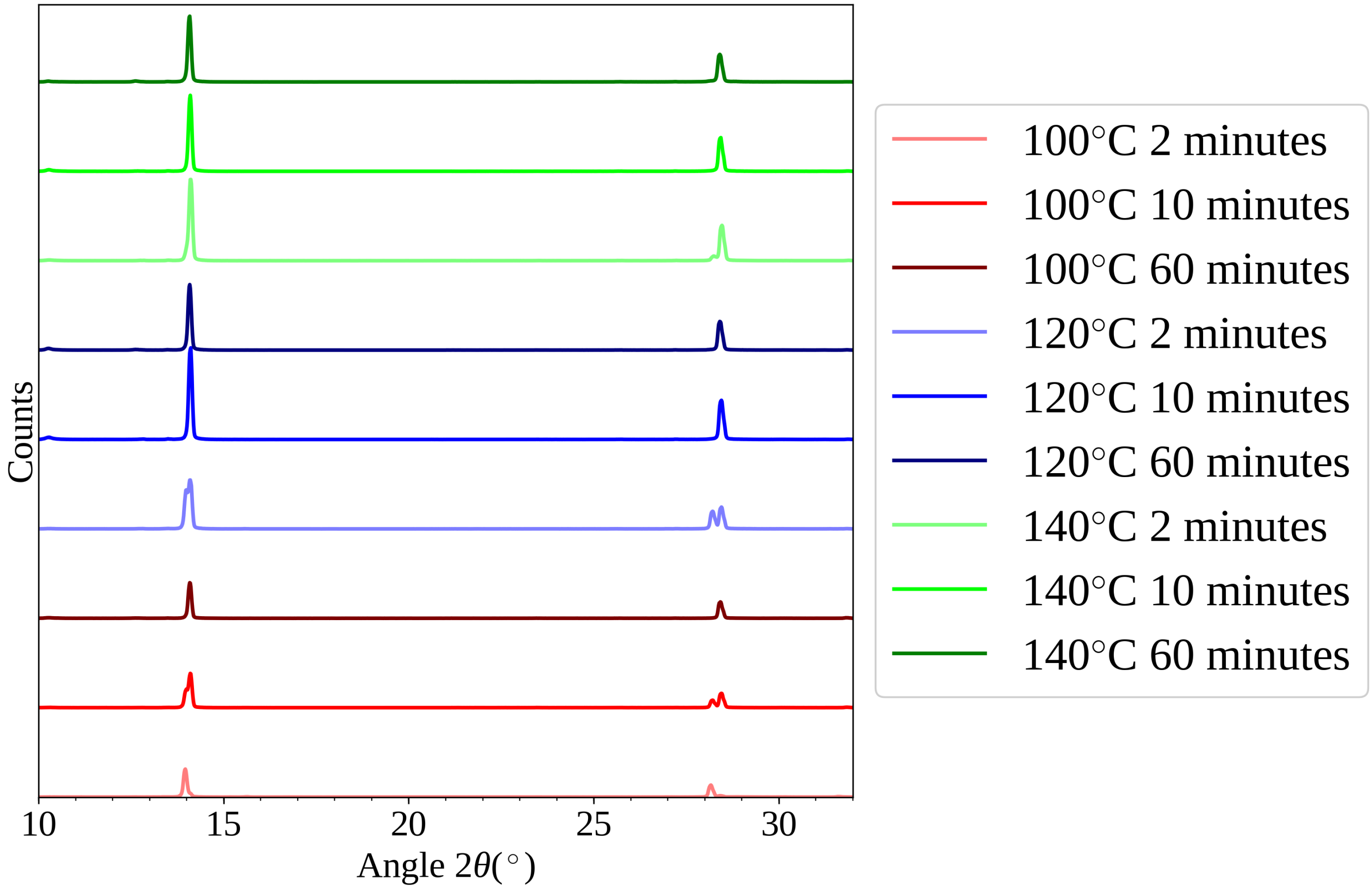

Figure S10: X-ray diffraction spectra of spin coated perovskite films on PEDOT coated ITO coated glass substrates with various drying times and temperatures.

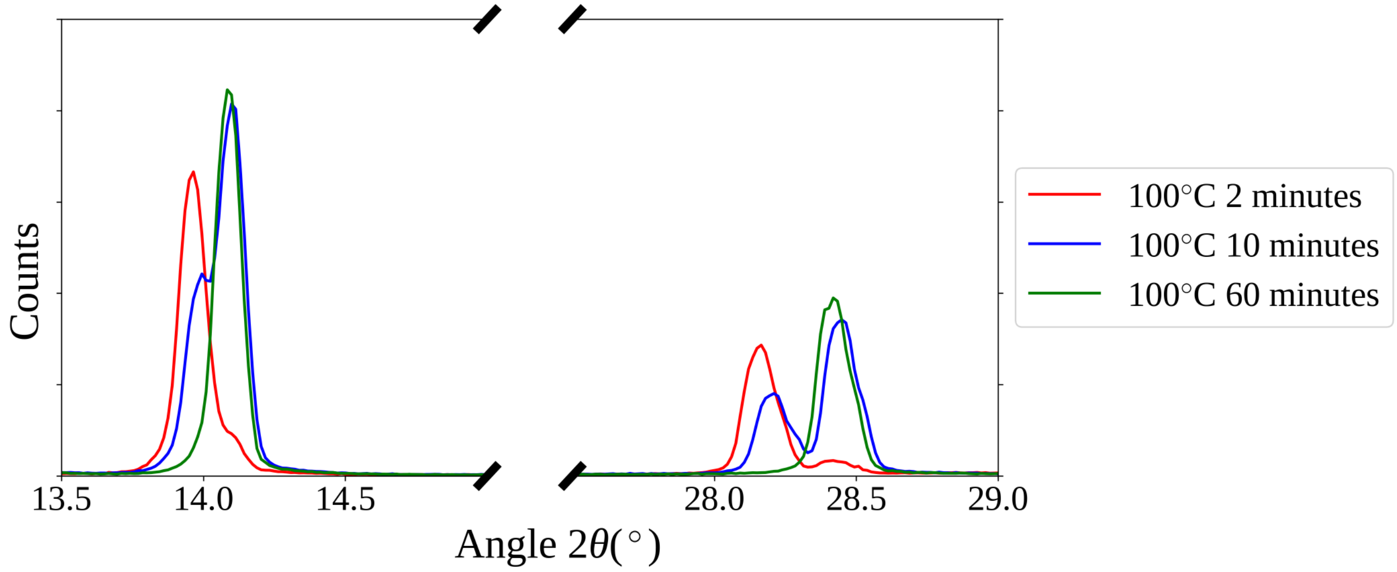

Figure S11: X-ray diffraction spectra of spin coated perovskite films on PEDOT coated ITO coated glass substrates dried at 100°C for various times.

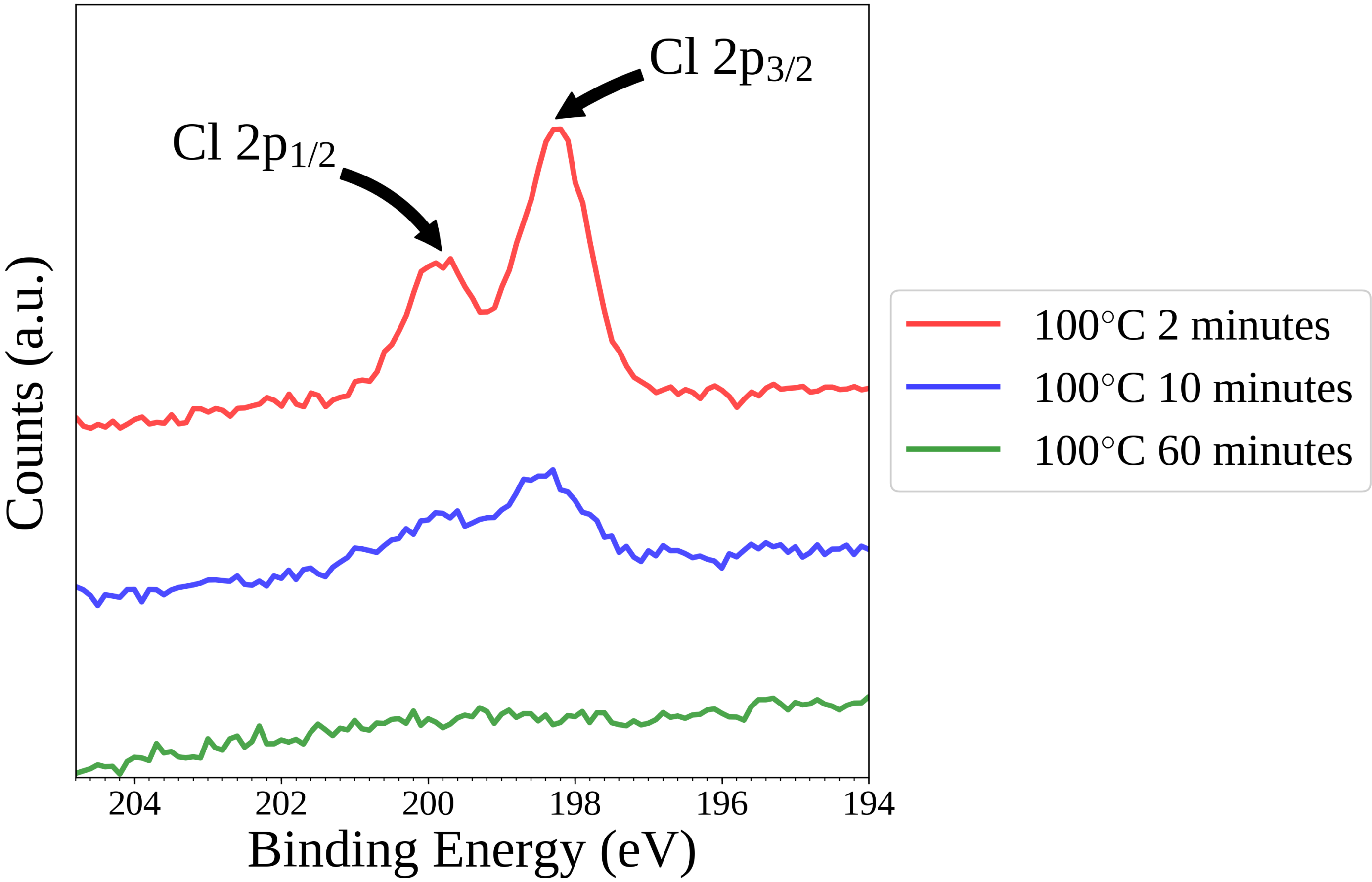

Figure S12: X-ray photoelectron spectroscopy spectra showing the change in the chloride $2p_{3/2}$ and $2p_{1/2}$ peaks for perovskite films dried at 100°C for various times.

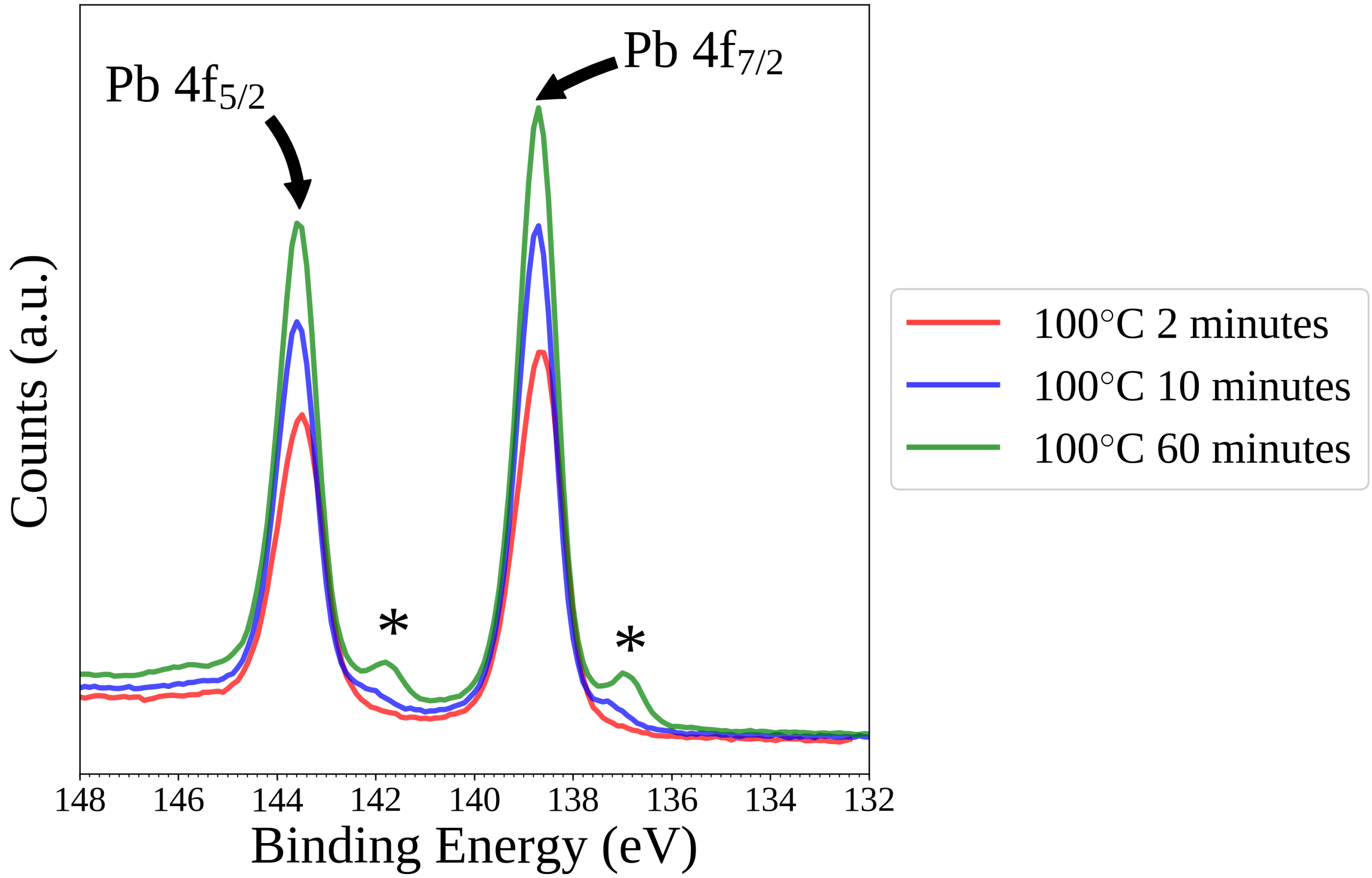

Figure S13: X-ray photoelectron spectroscopy spectra showing the change in the lead $4f_{5/2}$ and $4f_{7/2}$ peaks for perovskite films dried at 100°C for various times.

Table S4: X-ray photoelectron spectroscopy elemental distribution data for perovskite films dried at various times and temperatures.

Temperature (°C)	Time (minutes)	Carbon	Chlorine	Iodine	Nitrogen	Oxygen	Lead(II)
100	2	33.9	2.2	32.6	16.2	5.2	9.9
	10	35.9	0.9	32.0	14.4	5.8	11.0
	60	33.3	0.0	34.8	11.5	6.3	14.1
120	2	25.8	1.3	40.4	17.8	2.4	12.2
	10	30.2	0.6	37.9	14.2	3.9	13.2
	60	28.4	0.0	39.5	12.2	3.9	16.0
140	2	31.0	0.8	37.2	14.7	3.8	12.5
	10	29.7	0.0	38.7	12.3	3.9	15.5
	60	30.5	0.0	37.7	8.9	5.5	17.3

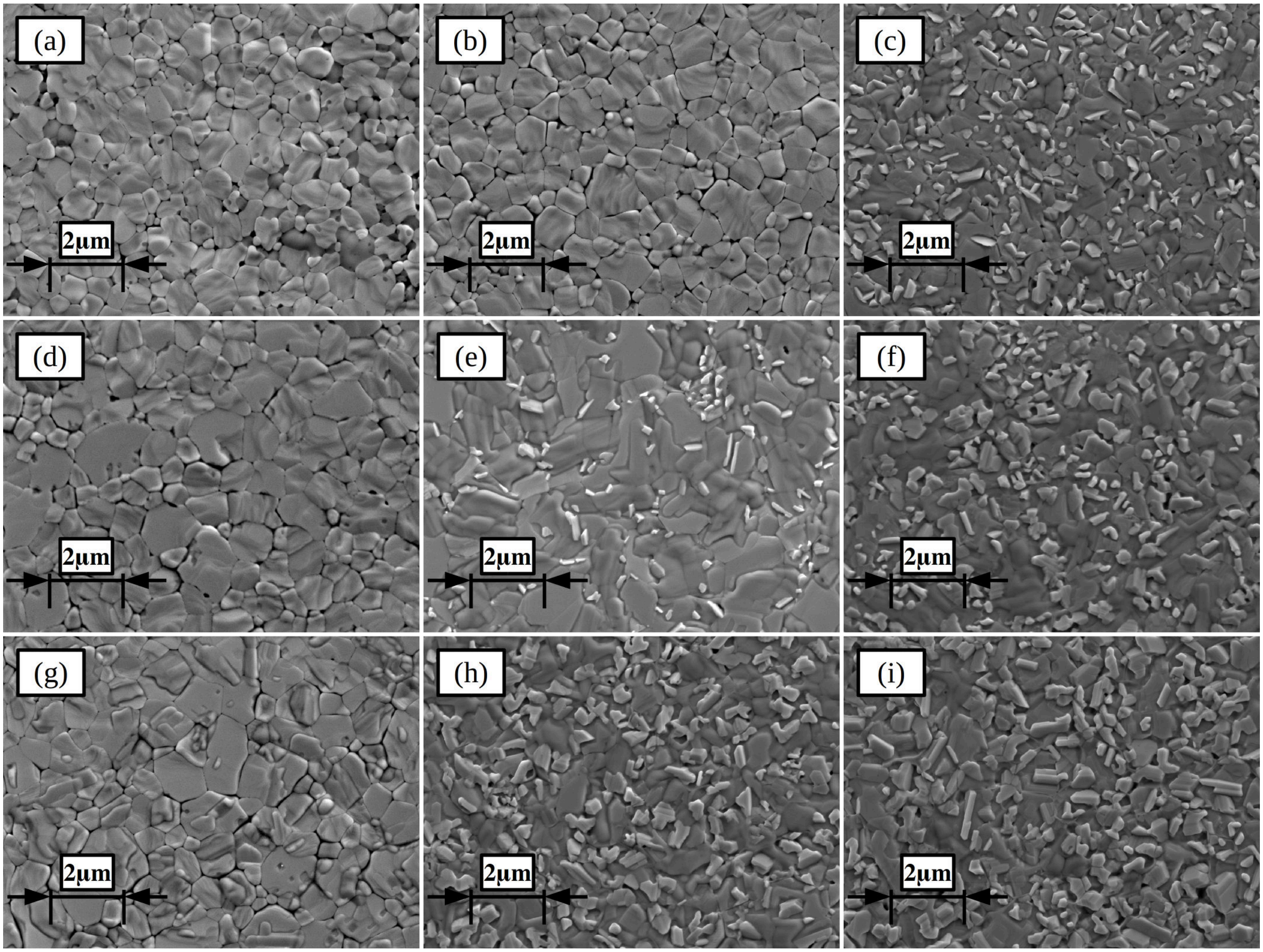

Figure S14: Top view SEM images of spin coated perovskite films on PEDOT coated ITO coated glass substrates with various drying temperatures and times. (a,b,c) 100°C, (d,e,f) 120°C, (g,h,i) 140°C with (a,d,g) 2 minutes, (b,e,h) 10 minutes, (c,f,i) 60 minutes. Inset scale bars represent 2 microns.

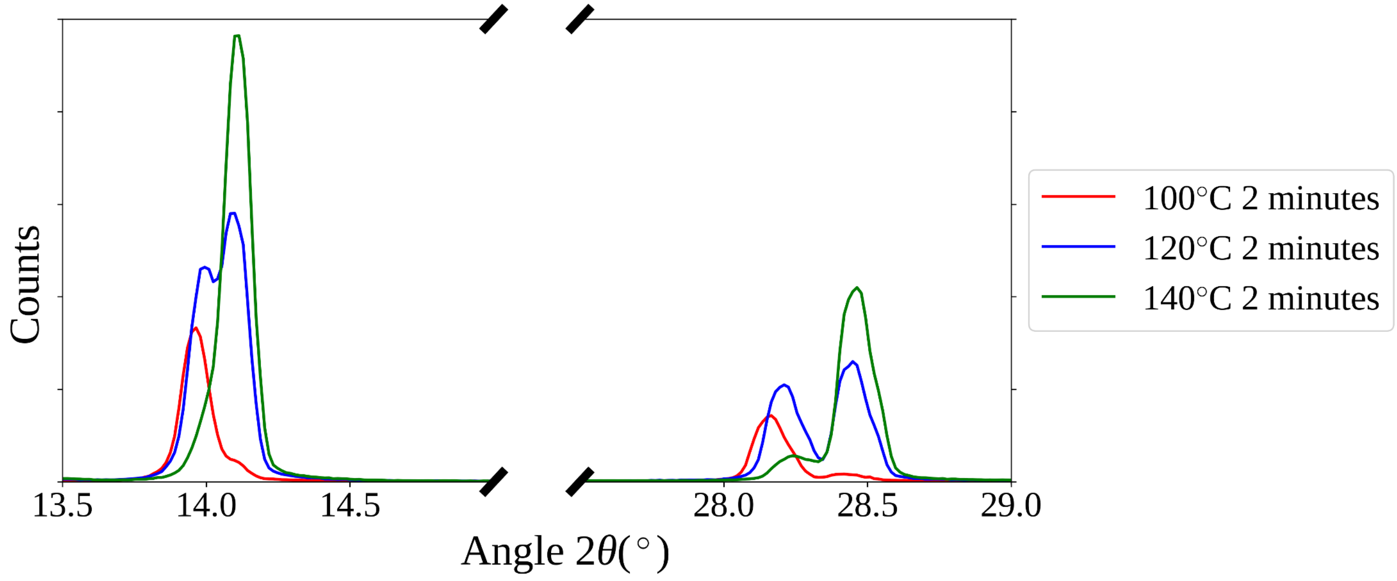

Figure S15: X-ray diffraction spectra of spin coated perovskite films on PEDOT coated ITO coated glass substrates dried at 100, 120 or 140°C for 2 minutes.

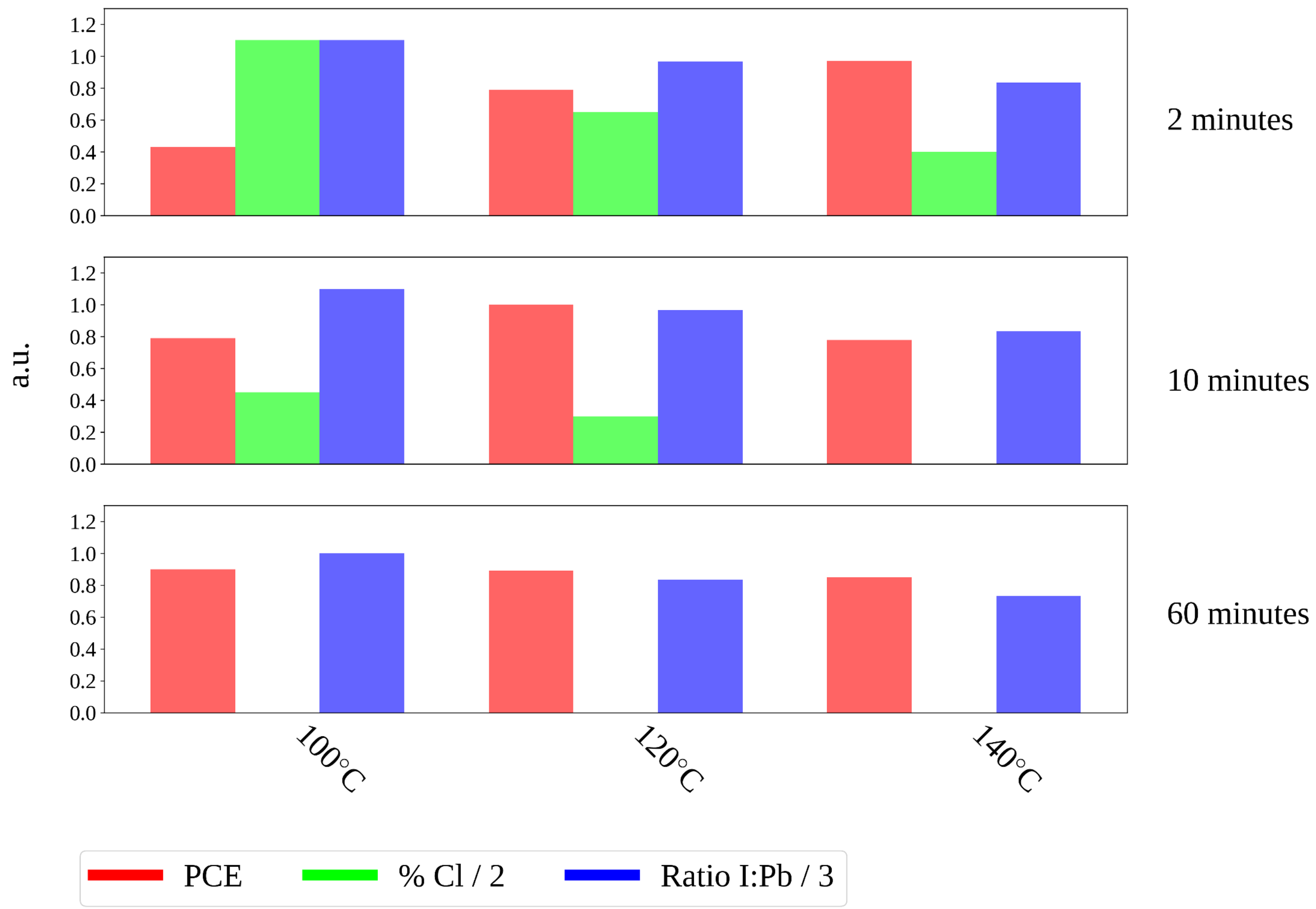

Figure S16: Bar charts of normalised to maximum PCE calculated from the average of reverse and forwards scan direction median values, % elemental surface chloride divided by two and ratio of I:Pb divided by three for perovskite films dried at various temperatures and times.

Table S5: Rheology of perovskite ink and contact angle on PEDOT:PSS film. Viscosity measurement reported for a shear rate of $2000 s^{-1}$.

Ink Concentration	Viscosity (mPa·s)	Surface Tension (mNm^{-1})	Contact Angle (°)
1.5M	0.82	29	<5
1.5M + $30 \mu l ml^{-1}$ $HCl_{(aq)}$	0.92	31	<5
1.0M	0.60	28	<5
1.0M + $20 \mu l ml^{-1}$ $HCl_{(aq)}$	0.66	34	<5

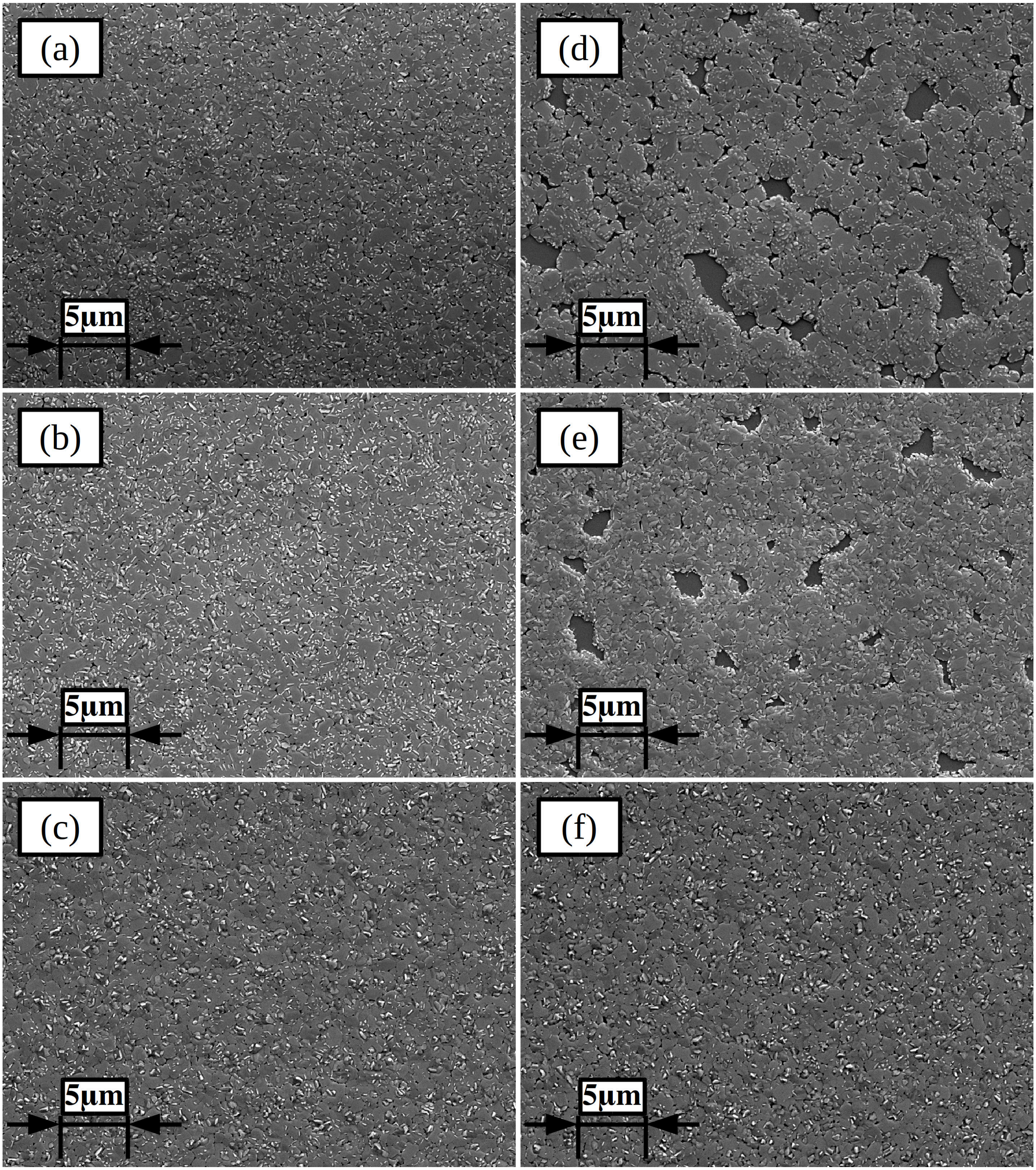

Figure S17: Top view SEM images of slot-die coated perovskite films on PEDOT coated ITO coated glass substrates with various air-knife flow rates, showing the difference between areas forming between solvent fronts and at the solvent front. (a,d) No air-knife applied, (b,e) air-knife flow rate of 25LPM, (c,f) flow rate of 50LPM. (a,b,c) Areas between solvent fronts, (d,e,f) areas at the solvent fronts. Inset scale bars represent 5 microns.

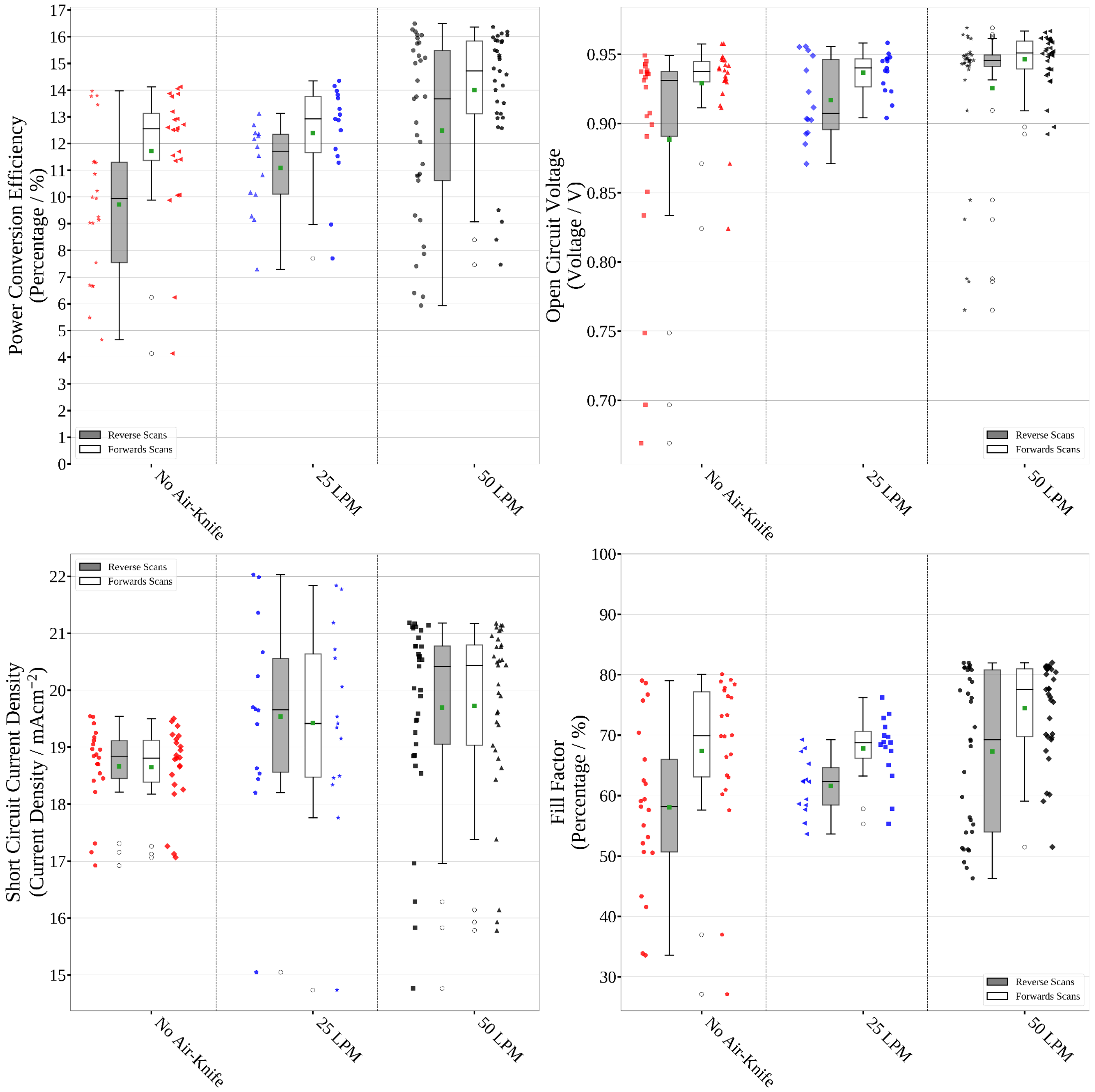

Figure S18: Box-plots of JV scan photovoltaic parameters for 0.09cm^2 cells prepared by slot-die coating of the perovskite film with application of various air-knife gas flow rates. The boxes represent the first and third quartiles, the horizontal black line the median, the upper whisker the data within 1.5 times the inter quartile range of the upper quartile and the lower whisker 1.5 times the inter quartile range of the lower quartile, green square the mean and open black dots outliers. The red, blue and black full markers represent the individual scan results for the adjacent scan direction and corresponding split.

Table S6: Median JV scan photovoltaic parameters of glass substrate devices prepared using different air-knife gas flow rates applied to the slot-die coated perovskite wet film.

Flow Rate (LPM)	Scan Direction	Voc (V)	Jsc ($mAcm^{-2}$)	FF (%)	PCE (%)
0	Forwards	0.94	18.8	70	12.6
	Reverse	0.93	18.8	58	9.9
25	Forwards	0.94	19.4	69	12.9
	Reverse	0.91	19.7	62	11.7
50	Forwards	0.95	20.4	78	14.7
	Reverse	0.95	20.4	69	13.7

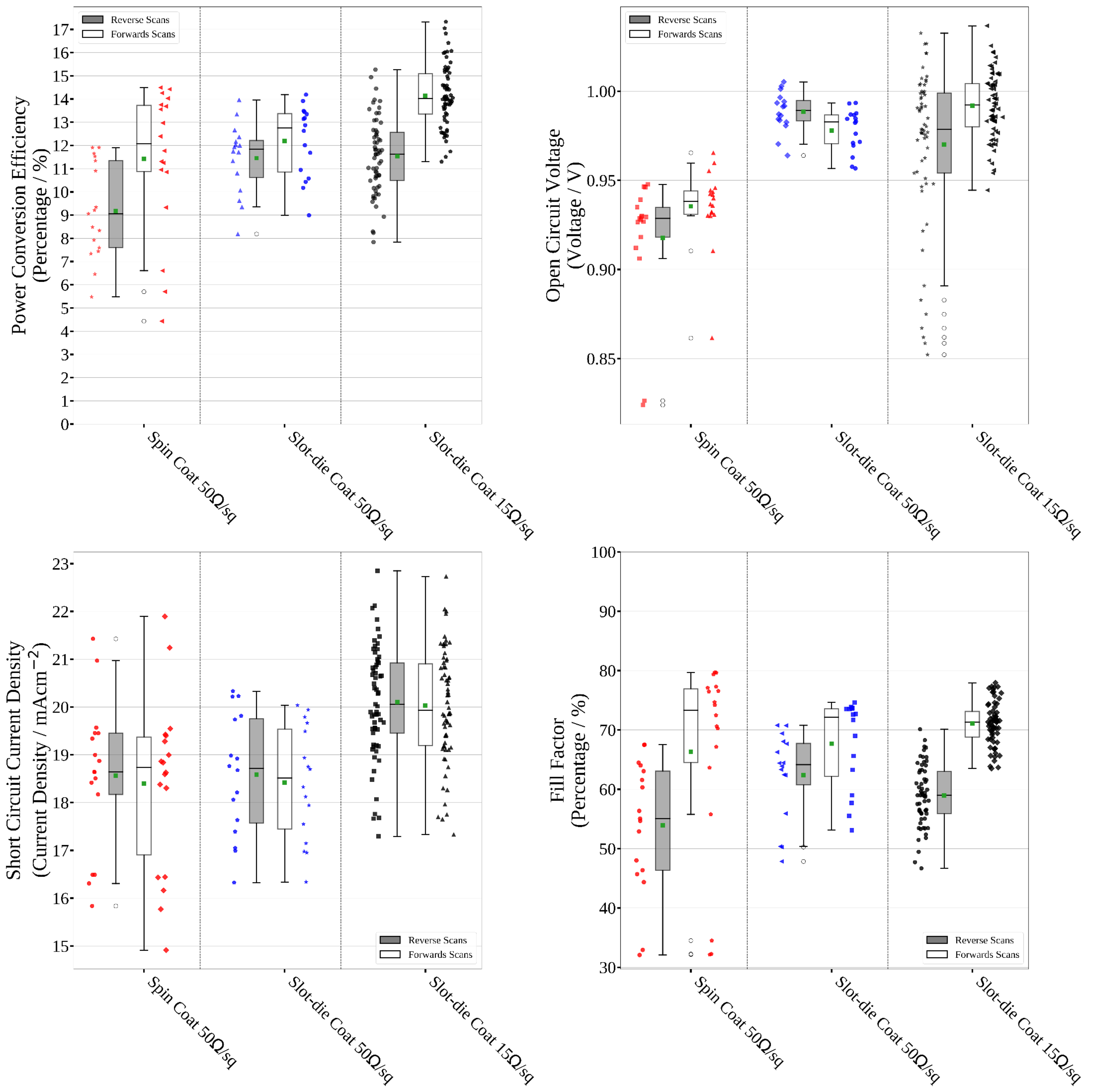

Figure S19: Box-plots of JV scan photovoltaic parameters for 0.09cm^2 cells with devices prepared on PET substrates with 15 or 50Ω/sq sheet resistance ITO. The boxes represent the first and third quartiles, the horizontal black line the median, the upper whisker the data within 1.5 times the inter quartile range of the upper quartile and the lower whisker 1.5 times the inter quartile range of the lower quartile, green square the mean and open black dots outliers. The coloured full markers represent the individual scan results for the adjacent scan direction and corresponding split.

References

[1] N. K. Noel, S. N. Habisreutinger, B. Wenger, M. T. Klug, M. T. Hrantner, M. B. Johnston, R. J. Nicholas, D. T. Moore and H. J. Snaith, *Energy Environ. Sci.*, 2017, **10**, 145–152.

[2] S. D. Stranks, G. E. Eperon, G. Grancini, C. Menelaou, M. J. P. Alcocer, T. Leijtens, L. M. Herz, A. Petrozza and H. J. Snaith, *Science*, 2013, **342**, 341–344.

[3] J. C. de Mello, H. F. Wittmann and R. H. Friend, *Advanced Materials*, 1997, **9**, 230–232.

[4] J. D. McGettrick, K. Hooper, A. Pockett, J. Baker, J. Troughton, M. Carnie and T. Watson, *Materials Letters*, 2019, **251**, 98 – 101.

[5] *NIST X-ray Photoelectron Spectroscopy Database, Version 4.1 (National Institute of Standards and Technology, Gaithersburg, 2012)*, http://srdata.nist.gov/xps/.

[6] R. Lindblad, N. K. Jena, B. Philippe, J. Oscarsson, D. Bi, A. Lindblad, S. Mandal, B. Pal, D. D. Sarma, O. Karis, H. Siegbahn, E. M. J. Johansson, M. Odelius and H. Rensmo, *The Journal of Physical Chemistry C*, 2015, **119**, 1818–1825.
